# Lessons Learned From a Delayed‐Start Trial of Modafinil for Freezing of Gait in Parkinson's Disease

**DOI:** 10.1002/acn3.70276

**Published:** 2025-12-08

**Authors:** Tuhin Virmani, Lakshmi Pillai, Reid D. Landes, Aliyah Glover, Shannon Doerhoff, Jennifer Kleiner, Mitesh Lotia, Rohit Dhall, Edgar Garcia‐Rill

**Affiliations:** ^1^ Department of Neurology University of Arkansas for Medical Sciences Little Rock Arkansas USA; ^2^ Department of Biomedical Informatics University of Arkansas for Medical Sciences Little Rock Arkansas USA; ^3^ Department of Biostatistics University of Arkansas for Medical Sciences Little Rock Arkansas USA; ^4^ Department of Psychiatry University of Arkansas for Medical Sciences Little Rock Arkansas USA; ^5^ Center for Translational Neuroscience University of Arkansas for Medical Sciences Little Rock Arkansas USA

**Keywords:** clinical trial, delayed‐start design, freezing of gait, Parkinson's disease, video‐based gait quantification

## Abstract

**Objective:**

Freezing of gait (FOG) in people with Parkinson's disease (PwPD) is debilitating and has limited treatments. Modafinil modulates beta/gamma band activity in the pedunculopontine nucleus (PPN), like PPN deep brain stimulation. We therefore tested the hypothesis that Modafinil would improve FOG in PwPD.

**Methods:**

PwPD with FOG were randomized to early‐start (24 weeks modafinil) or delayed‐start (12 weeks each, placebo then modafinil) of oral modafinil 50 mg, followed by a 2‐week washout for both arms. Primary outcomes were change in OFF‐levodopa stride length and FOG questionnaire scores; secondary outcomes were change in motor Unified Parkinson's Disease Rating Scale (UPDRS), sleep and quality‐of‐life scores, and post hoc outcome was change in percent freezing time (%FT).

**Results:**

Early‐ (*n* = 12) and delayed‐start (*n* = 9) group participants were well matched for age, OFF‐levodopa motor UPDRS and FOG‐Q scores. Primary and secondary outcomes did not reach statistical significance. Limiting the analysis to study completers with quantifiable visualized freezing at the initial visit and collapsing the two study arms (*n* = 11), %FT trended to improvement with 50 mg modafinil in 8/11 participants (*p* = 0.15) and worsening after 2 weeks washout in 9/11 participants (*p* = 0.09). A future cross‐over study with 50 participants would have 0.80 power to detect a 0.5 standard‐deviation improvement in %FT.

**Interpretation:**

For future therapeutic trials, selecting PwPD with moderate, quantifiable FOG and utilizing appropriate outcome measures like %FT will improve the ability to identify intervention effects. Even for short trials, analyses must account for gait decline. An FOG outcome measure that requires less analysis time burden than video quantification, and the ability for at‐home monitoring is needed.

## 1 Introduction

Freezing of gait (FOG) is a debilitating complication of Parkinson's disease (PD). It is defined as a “brief, episodic absence or marked reduction of forward progression of the feet despite the intention to walk,” often described as the feeling of the feet “sticking to the ground” for several seconds during attempted active movement [1, 2]. FOG occurs commonly during turning or when initiating gait (start–hesitation) but can also occur mid‐stride, at destination, in doorways, in tight spaces and with a time pressure to complete a task [3, 4]. Freezing is worsened in dual‐task paradigms where attention is distracted from the task of walking [5, 6]. Freezing leads to decreased mobility, increased rates of falls, and subsequent fear of falling, which further limits mobility [7, 8, 9] ultimately resulting in a significant worsening of quality of life [10, 11].

Currently, treatment options are limited to exercise and physical therapy, especially in people whose freezing does not respond to levodopa [12, 13, 14, 15]. Methylphenidate inhibits presynaptic dopamine [16] and the noradrenaline transporter. High‐dose methylphenidate (1 mg/kg/day) is shown to reduce step counts in PD patients with subthalamic Deep Brain Stimulation (DBS), but its use is limited by cardiac side effects [17]. Stimulation of the pedunculopontine nucleus (PPN) at beta and gamma band frequencies (40–60 Hz) has been shown to improve FOG in animal models as well as in PD [18, 19, 20, 21, 22], possibly by releasing pre‐prepared motor programs [23].

Modafinil, an atypical stimulant approved for the treatment of narcolepsy, has a number of putative sites of action throughout the brain [24], among which is an increase in neuronal electrical coupling and maintaining beta and gamma band activity in the PPN [25, 26, 27]. The PPN, located at the mesopons, is extensively reciprocally connected with the cortex, basal ganglia, thalamus, and spinal cord [22], making it a potential modulation target. PPN neurons fire at beta/gamma frequencies during waking [28]. These oscillations are mediated by high‐threshold voltage‐dependent N‐ and P/Q‐type calcium channels [29], and are blocked by KN‐93 (a CAMKII activation inhibitor), suggesting that at least some cells produce oscillations through the CAMKII pathway. Modafinil increases neuronal electrical coupling through a CAMKII pathway [25, 30], thereby promoting gamma frequency activity. Increased gamma band activity promotes activation of ascending “wake state” pathways and descending locomotor pathways [31, 32].

Through its promotion of gamma oscillations in the PPN, we therefore hypothesized that Modafinil would improve gait in PD, thereby providing a useful and safer pharmacologic alternative to surgical PPN DBS.

## 2 Methods

### 2.1 Standard Protocol Approvals, Registrations, and Patient Consents

Participants were recruited from the Movement Disorders Clinic at the University of Arkansas for Medical Sciences (UAMS) between September 2014 and December 2019. Inclusion criteria were (1) age 50–90 years, (2) diagnosis of idiopathic PD on UK brain bank criteria [33], (3) presence of FOG based on objective assessment by the movement disorders neurologist (TV), (4) Freezing of Gait Questionnaire (FOG‐Q) [34] score > 8, and (5) stable PD therapy (including medications and stimulation) for 3 months prior to enrollment.

Exclusion criteria were: (1) antidopaminergic medication use within 1 year from the date of enrollment (excluding quetiapine < 100 mg/day or clozapine); (2) anticipated requirement of PD medication adjustment over the 6‐month trial period; (3) history of allergic reactions to Modafinil or Armodafinil; (4) uncontrolled intercurrent illness including, but not limited to, ongoing or active infection, symptomatic congestive heart failure, unstable angina pectoris, cardiac arrhythmia, mitral valve prolapse, left ventricular hypertrophy, chronic obstructive pulmonary disease, known malabsorption syndromes, renal disease or hepatic disease, or psychiatric illness/social situations that would limit compliance with study requirements; (5) individuals who were pregnant or breastfeeding; and (6) non‐English‐speaking individuals who were unable to complete assessments in English or follow English instructions.

The study protocol (Supporting Information) was approved by the UAMS Institutional Review Board (UAMS IRB# 002734) and pre‐determined plans for participant randomization, outcome measures, and statistical analysis are outlined in sections below. A United States Food and Drug Administration (FDA) Investigational New Drug application (IND: 135059) was approved based on the submitted protocol. The study was registered on the ClinicalTrials.gov website (NCT03083132). Written informed consent was obtained from each participant prior to initiating study procedures. The study was conducted in accordance with the guidelines of the Declaration of Helsinki and International Council for Harmonization (ICH) Good Clinical Practice (GCP) standards.

### 2.2 Participant Randomization

**FIGURE 1:**
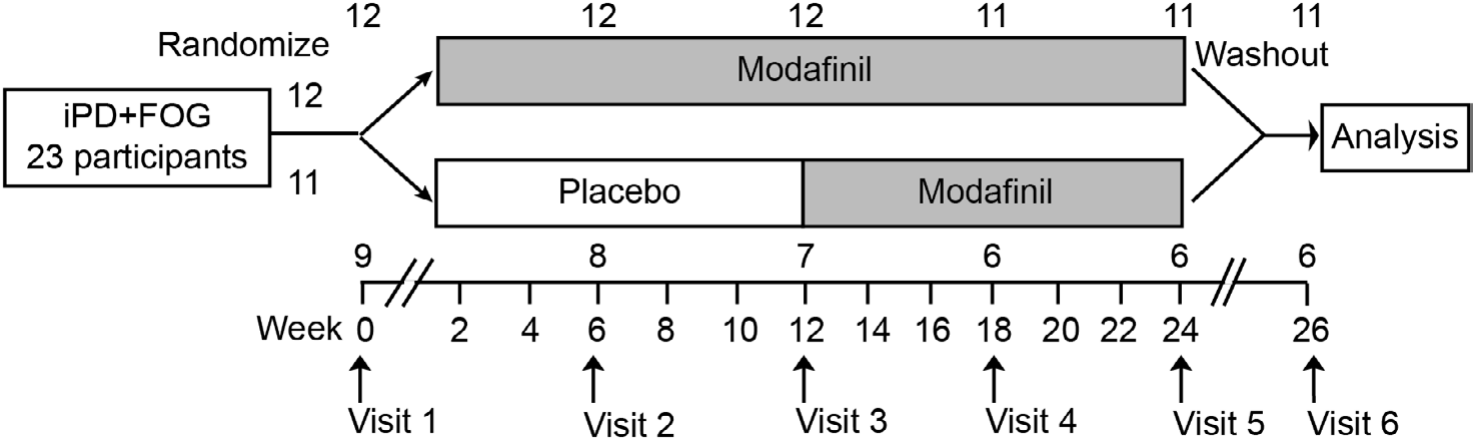
Trial design. The blinded, randomized, placebo‐controlled, delayed‐start clinical trial design is shown, including the timing of evaluations performed. The axis shows study duration in weeks while the numbers indicate the participants remaining in each group at the different stages of the trial.

After enrollment, participants were randomized to one of two study arms: (1) an early‐start arm, where participants received modafinil 50 mg starting the day after their initial enrollment visit for a total of 24 weeks, or (2) a delayed‐start arm, where participants received 12 weeks of placebo followed by 12 weeks of modafinil (Figure 1). Both arms underwent a 2‐week washout before final assessments at 26 weeks. The randomization log was developed and maintained by the UAMS research pharmacy, randomizing participants 1:1 sequentially to early‐start or delayed‐start modafinil. The investigators remained blinded to participant study arm until raw data analysis required for the analysis of outcomes was completed.

### 2.3 Study Drug and Placebo

Modafinil 50 mg oral capsules and placebo capsules were produced and dispensed by the UAMS research pharmacy in identical gelatin capsules with microcrystalline cellulose as filler. Participants were provided a 6 weeks' supply of modafinil or placebo at a time by the pharmacy. Participants kept a pill diary documenting dosing compliance and returned pill containers at each visit. Participants unable to provide this log signed a form stating their compliance with the study dosing regimen. Overall diary compliance was 95%. Seventeen percent missed doses, with 1–7 returned pills in a 42‐day period. Two participants missed 7 doses; both in the first 6 weeks with 1 in each study arm.

### 2.4 Pre‐Defined Outcome Measures

#### 2.4.1 *Primary outcome measures* pre‐defined in the protocol were

1. freezing severity using the Giladi Freezing of Gait Questionnaire (FOG‐Q) score [34] and
2. gait stride length during continuous gait.

#### 2.4.2 *Secondary outcome measures* pre‐defined in the protocol were

1. OFF‐state Unified Parkinson's Disease Rating Scale (UPDRS) [35] part III motor scores;
2. quality of life measured using the Parkinson's Disease Questionnaire −39 (PDQ‐39) [36] scale;
3. sleep quality (due to PPN modulation by modafinil) measured using the Epworth sleepiness scale (ESS) [37] and rapid eye movement (REM) REM Sleep Behavior Disorder Screening Questionnaire (RBD‐Q) [38];
4. adverse events associated with modafinil in the early and delayed‐start arms.

#### 2.4.3 *Exploratory Outcome Measures* Pre‐Defined in the Protocol Were

1. Cognitive function measured using the Montreal Cognitive Assessment (MoCA) [39], the Frontal Assessment Battery (FAB) [40], and the Scales for Outcome in Parkinson's disease—Cognition (SCOPA‐COG) [41] and
2. objective gait measures (such as stride width, stride velocity, swing and stance phase percent, etc.) during continuous gait.

The primary, secondary, and exploratory outcomes (except cognitive measures) were recorded at baseline, 6, 12, 18, and 24 weeks, and after drug washout at 26 weeks. Cognitive measures were recorded at baseline, 12, 24 and 26 weeks. A priori, all outcomes, except gait, were planned to be compared between early‐ and delayed‐start arms between baseline and 12 weeks (baseline, 6 and 12 weeks). For gait outcomes, comparison of the slopes of change between early‐ and delayed‐start arms through 12 weeks was planned. Within the delayed‐start arm, we originally planned to compare slope differences between the first 12 weeks (baseline, 6 and 12 weeks) to the next 12 weeks (12, 18 and 24 weeks) for gait outcomes; however, we do not report this comparison here. For conformity among reported outcomes, baseline to 12 weeks assessment results between early‐ and delayed‐start arms were compared with the same statistical model.

We decided a priori that participants completing at least 6 weeks of follow‐up in either arm were to be included in the primary and secondary outcomes analysis. One participant in the delayed‐start arm dropped out after the baseline visit and one after 6 weeks; all others completed at least 12 weeks.

### 2.5 Dopaminergic Medication State

All participants performed motor UPDRS and gait assessments in the relative levodopa OFF‐state withholding levodopa and dopamine agonists for at least 12 h as in prior studies [42, 43, 44]. Participants with DBS were evaluated with the stimulator off. Study drug or placebo was taken by participants in the morning prior to study assessments. The Levodopa Equivalent Daily Dose (LEDD) for each participant at each visit was calculated based on prior reported estimations of levodopa bioavailability [45], and equivalency of dopamine agonist and MAO‐Inhibitor dosages [46].

### 2.6 Gait Analysis

While multiple gait assessments were defined in the original protocol, due to significant freezing in some participants, only three assessments were consistently performed and analyzed in the levodopa OFF‐state (and DBS OFF‐state) and were adequate for analysis of pre‐defined outcomes. (1) Participants were instructed, using similar verbiage and visual demonstration, to walk at their comfortable pace and make normal 180° pivot turns, at or before cones placed approximately 1′ from each end of a 20′ × 4′ instrumented gait mat (Zeno walkway, Protokinetics Inc., USA) (single‐task or ST‐gait). Guidance was not given on turn direction. The goal was to obtain 7 turns for each participant. (2) Participants repeated the ST‐gait task with the dual‐task condition counting backwards while walking (DT‐gait). (3) A timed‐up‐and‐go (TUG) task standing up from a chair placed at one end of the mat, walking to the other end, turning, walking back, and sitting down, was performed 4 times.

Gait data were collected and analyzed as previously published using Protokinetics Movement Analysis Software (PKMAS) [13, 47, 48]. For the pre‐defined gait outcomes, features were extracted from ST‐gait continuous gait segments by excluding turns.

### 2.7 Post hoc Analysis

#### 2.7.1 Gait analysis

Turn segments were isolated from the ST‐gait task using protocols we previously published [49]. TUG task measures were manually defined by observing concurrent video and gait pressure trace recordings using PKMAS software. For each visit, the mean and percent coefficient of variation (CV) were analyzed for 23 spatiotemporal gait parameters for straight walking segments from ST‐ and DT‐gait tasks, 19 parameters for turn segments from the ST‐gait task and 6 parameters specific to TUG (Table S1).

#### 2.7.2 Freezing episode analysis

Analysis of freezing episode time from video recordings is becoming the gold standard for evaluation of therapeutic response in FOG trials [50, 51, 52]. A movement disorders neurologist (T.V.) therefore identified clinical freezing episodes [1] for OFF‐state (including DBS‐OFF) ST‐gait including both straight and turn segments [53], blinded to treatment arm. Briefly, time‐locked ST‐gait video recordings perpendicular and longitudinal to the direction of walking were reviewed. Freezing episodes were further reviewed frame‐by‐frame to identify episode start and end times. The total freezing time (FT) and the percentage of total walk‐time a participant spent freezing (%FT) were calculated for individuals at baseline, 12, 24, and 26 weeks.

### 2.8 Statistical Analysis

The primary and secondary outcome measures from weeks 0 through 12 were analyzed in a linear mixed model accounting for treatment, time of observation (0, 6 or 12 weeks), and the treatment × time interaction; participant was treated as a random effect. A first‐order autoregressive correlation structure was used to model the within‐participant correlation of repeated measures. For all participants, the treatment assigned at randomization was used as the individual's treatment in the statistical analysis; that is, we used an intent‐to‐treat analysis. One participant who dropped out after 6 weeks was included in the analysis as planned. The mean change from baseline to 12 weeks was estimated for each treatment group, and statistically compared between treatment groups within the linear mixed model framework; that is, treatment × time interaction. The standard deviation (SD) was estimated with the root mean square error from the linear mixed model. Error degrees of freedom were estimated with the Kenward‐Roger 2009 method. A Wilcoxon‐paired‐rank test was used for the post hoc %FT, collapsed groups analysis.

Based on our pre‐study power analysis we intended to complete 10 participants in each study arm. With such, we would have between 0.70 and 0.90 power to detect if the 12‐week change from baseline in the early‐start arm (on modafinil) was 1.75 SD greater than the 12‐week change in the delayed‐start arm (on placebo) on a two‐sided 0.05 significance level test. The SD is the within‐group SD, assumed constant across time. The power depends on the within‐individual correlation (assumed to be first‐order autocorrelation): within‐individual correlations of 0.20, 0.35, and 0.55 corresponded to power of 0.70, 0.80, and 0.90, respectively. Based on SD estimates from previous researchers in similar settings [54], the FOGQ (the primary outcome) has a SD of 3.58, implying that we would have had power to detect a difference in 12‐week changes of 6.27 (=1.75 × 3.58) units.

### 2.9 Data Sharing

The data that support the findings of this study will be made openly available on figshare with an associated DOI.

## 3 Results

### 3.1 Participant Enrollment and Demographics

**FIGURE 2:**
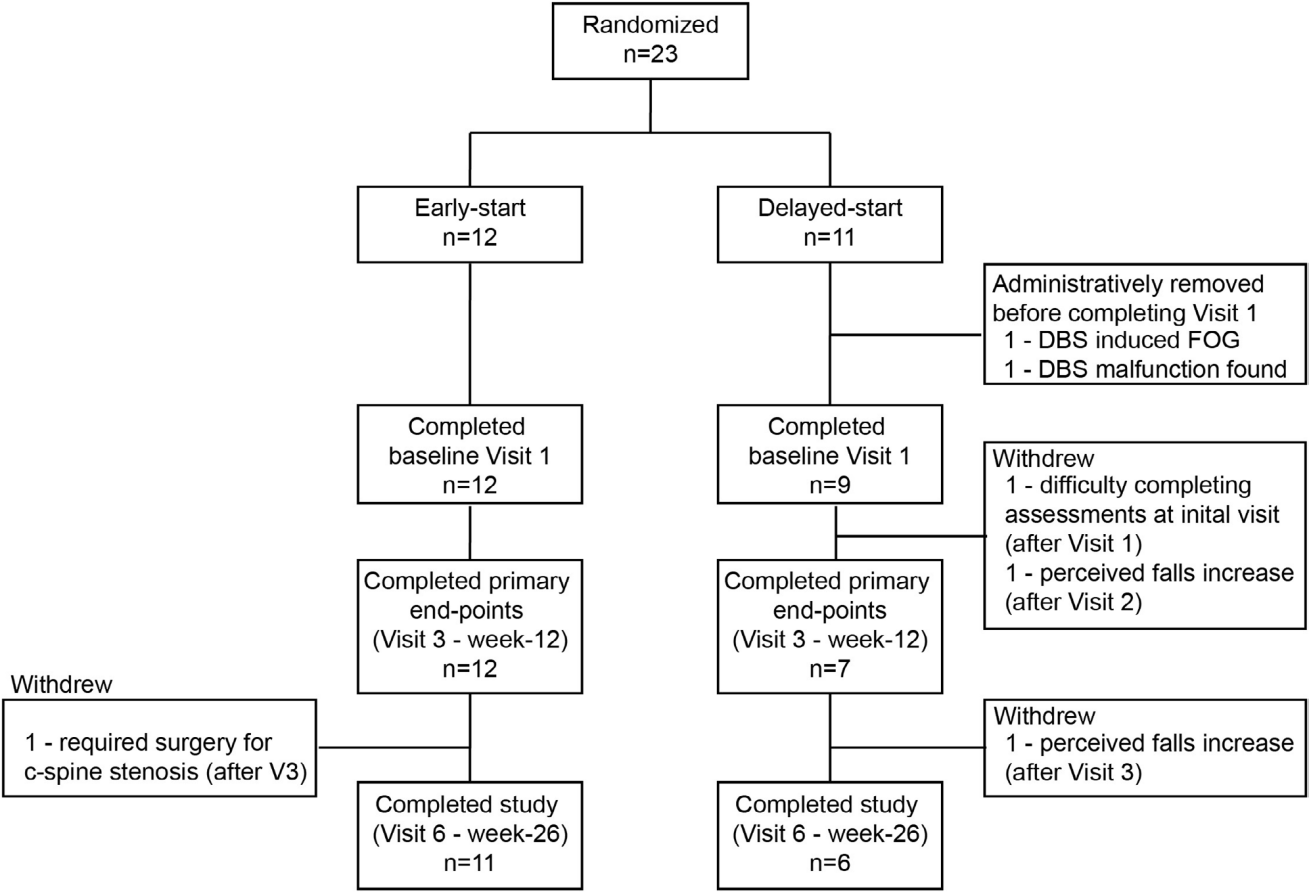
CONSORT Flowsheet. The flow of participants through the trial and the reasons for drop‐out are demonstrated.

Twenty‐three participants were enrolled and randomized to early‐ (*n* = 12) or delayed‐start (*n* = 11) of modafinil (Figure 2) with the goal of completing 10 participants in each group. Two participants were administratively removed after blinded randomization (both in the delayed‐start arm). In one participant, FOG was deemed to be a stimulation side effect as it resolved when DBS was turned OFF. In the other participant, a DBS malfunction was discovered that needed addressing.

Twenty‐one participants completed the baseline visit, with the groups well matched for participant age, Hoehn and Yahr staging, OFF‐state motor and total UPDRS scores, FOG‐Q scores and cognitive function (Table 1). Disease duration in the delayed‐start group was 3.8 years longer than the early‐start group; a 0.6 SD spread (Table 1).

**TABLE 1:** Modafinil study Demographics and baseline assessments.

Feature	Early‐start (*n* = 12)	Delayed‐start (*n* = 9)
Age (years)	71.6 ± 6.3	72.9 ± 5.3
Disease duration (years)	9.8 ± 5.1	13.6 ± 7.4
Sex (F/M)	3/9	3/6
Hoehn and Yahr stage	3.5 ± 0.8	3.3 ± 0.9
Completed visit 3 (12 weeks)—randomized	12	7
Completed visit 5 (24 weeks)—all on modafinil	11	6
Completed visit 6 (26 weeks)—washout of modafinil	11	6
OFF‐state Motor‐Unified Parkinson's Disease Rating Scale (UPDRS)	37.7 ± 5.9	38.0 ± 8.3
OFF‐state Total‐UPDRS	65.3 ± 10.5	64.6 ± 14.0
ON‐state Motor‐UPDRS	32.7 ± 8.8	30.5 ± 8.8
ON‐state Total‐UPDRS	57.4 ± 13.7	52.8 ± 17.2
Freezing of Gait Questionnaire (FOG‐Q) total score	13.8 ± 2.5	14.4 ± 1.7
FOG‐Q freezing sub‐score (items 3–6)	8.7 ± 1.7	9.4 ± 1.2
FOG duration (years)	4.5 ± 2.8	5.3 ± 4.3
Montreal Cognitive Assessment score (MoCA)	21.8 ± 4.7	23.4 ± 4.6
Scales for Outcomes in Parkinson's‐cognition score (SCOPA‐COG)	14.3 ± 2.3	14.6 ± 2.4
Frontal Assessment Battery score (FAB)	18.4 ± 6.2	22.0 ± 4.8
Parkinson's Disease Questionnaire (PDQ‐39) score	59.8 ± 21.1	60.7 ± 21.3
Apathy Evaluation Scale score (AES)	33.3 ± 5.2	31.1 ± 6.5
Epworth Sleepiness Scale score (ESS)	10.3 ± 2.9	10.1 ± 4.6
REM Behavior Disorder Questionnaire score (RBD‐Q)	5.1 ± 3.5	5.3 ± 2.9
Daily levodopa dose (mg/day)	923 ± 494	1085 ± 401
Participants with Deep Brain Stimulators (DBS)	1	2 (1 withdrew after 6‐week visit)

*Note:* Summaries are expressed as counts or means ± standard deviations.

Two participants in the delayed‐start group withdrew prior to completing the 12‐week visit (Figure 2) while one delayed‐start participant withdrew after the 12‐week visit due to a reported increase in falls on placebo (Figure 2). In the early‐start arm, only one participant withdrew after completing the 12‐week visit due to a diagnosis of c‐spine stenosis requiring surgical intervention (Figure 2).

### 3.2 Pre‐Defined Outcome Measures

#### 3.2.1 Primary Outcome Measures

1. *Change in FOG‐Q:* The mean change from baseline FOG‐Q score after 12 weeks on Modafinil was +0.67 points, which was 1.81 points higher (95% CI: −0.73, 4.35) than the same mean change for those on Placebo for 12 weeks (Table 2). Thus, there was no evidence that Modafinil improved subjectively‐reported FOG more than in changes experienced by those on Placebo.
2. *Change in stride length:* The mean change from baseline stride length after 12 weeks on Modafinil was −5.74 cm, which was 5.36 cm less (95% CI: −7.31, 18.03) than the same mean change for those on Placebo for 12 weeks (Table 2). Thus, there was no evidence to suggest that Modafinil changes stride length during ST‐gait.

**TABLE 2:** Modafinil primary, secondary, and exploratory outcome summary statistics, estimated from the linear mixed model.

	Delayed‐start Placebo (*n* = 7)		Early‐start Modafinil (*n* = 12)		Estimated correlation (week 0–12)
	0 week	12 week	0 week	12 week	
Primary outcomes					
FOG‐Q score	14.57 ± 2.84	13.43 ± 2.84	13.75 ± 2.84	14.42 ± 2.84	0.74
Stride‐length (cm)*	90.13 ± 26.64	89.74 ± 26.64	69.78 ± 26.64	64.04 ± 26.64	0.93
Secondary outcomes					
UPDRS‐III	39.00 ± 3.43	34.36 ± 3.43	37.71 ± 3.43	37.25 ± 3.43	0.85
PDQ‐39	65.7 ± 22.0	63.3 ± 22.0	59.8 ± 22.0	62.6 ± 22.0	0.88
RBD‐Q	5.42 ± 1.54	3.57 ± 1.54	5.08 ± 1.54	5.58 ± 1.54	0.72
ESS	9.00 ± 2.15	8.71 ± 2.15	10.33 ± 2.15	10.25 ± 2.15	0.66
Exploratory outcomes					
MoCA	25.1 ± 4.5	25.1 ± 4.5	21.8 ± 4.5	21.3 ± 4.5	0.91
FAB	14.6 ± 3.0	15.1 ± 3.0	14.3 ± 3.0	12.8 ± 3.0	0.89
SCOPA	23.6 ± 2.1	24.3 ± 2.1	18.4 ± 2.1	17.3 ± 2.1	0.91

*Note:* Results are mean ± standard deviation, where the model‐estimated standard deviation is the root mean square error from the model.

Abbreviations: ESS, Epworth Sleepiness Scale; FAB, Frontal Assessment Battery; FOG‐Q, freezing of gait questionnaire; MoCA, Montreal Cognitive Assessment; PDQ‐39, Parkinson's Disease Questionnaire; RBD‐Q, REM Sleep Behavior Disorder Screening Questionnaire; SCOPA‐Cog, Scales for Outcome in Parkinson's disease—Cognition; UPDRS‐III, Unified Parkinson's Disease Rating Scale part 3–motor.

* Single‐task gait.

#### 3.2.2 Secondary Outcome Measures

1. *Change in UPDRS part III motor score:* The mean change from baseline in OFF‐state UPDRS motor scores after 12 weeks on Modafinil was −0.46 points, which was 4.18 points more (95% CI: −0.40, 8.77) than the same mean change for those on Placebo for 12 weeks (Table 2).
2. *Change in PDQ‐39:* The mean change from the baseline PDQ‐39 score after 12 weeks on Modafinil was +2.83 points, which was 5.26 points more (95% CI: −5.12, 15.64) than the same mean change for those on Placebo for 12 weeks (Table 2).
3. *Change in sleep function:* The mean change from the baseline RBD‐Q score after 12 weeks on Modafinil was +0.50 points, which was 2.36 points more (95% CI: 0.30, 4.42) than the same mean change for those on Placebo for 12 weeks (Table 2). The mean change from the baseline ESS score after 12 weeks on Modafinil was −0.08 points, which was 0.20 points more (95% CI: −2.67, 3.07) than the same mean change for those on Placebo for 12 weeks.
4. *Adverse event profile:* Rash is a reported adverse event of modafinil, and one participant in the early‐start group developed a facial rash and chose to remain in the study. The participant was prescribed doxycycline by their PCP, and the rash improved, despite remaining on the study drug. Two participants attributed increased falls to the study drug, which is not an anticipated adverse event for modafinil. Both were in the delayed‐start arm of the study, and therefore on placebo at the time of withdrawal (6 and 12 weeks). Study‐unrelated adverse events were reported by all participants and are summarized in Table S2.

#### 3.2.3 Exploratory Outcomes

1. *Changes in other continuous ST‐gait features:* There were no significant differences between baseline and 12 weeks in the early‐ and delayed‐start arms.
2. *Change in cognitive measures:* The early‐start arm's decrease in MoCA scores did not statistically differ from the delayed‐start arm (early‐start −0.58 points, delayed‐start 0 points, 95% CI: −3.15, 1.98; *p* = 0.650) (Table 2). The early‐start arm's change in FAB was significantly lower than the delayed‐start arm's change by 2.07 points (early‐start −1.5 points, delayed‐start 0.57 points, 95% CI: −3.95, −0.19; *p* = 0.032). The early‐start arm's change in SCOPA‐Cog was lower than the delayed‐start arm's change by 1.80 points, but not significantly so (early‐start −1.08 points, delayed‐start 0.71 points, 95% CI: −4.62, 1.03; *p* = 0.207).

### 3.3 Post Hoc Analysis

#### 3.3.1 Changes in Other Gait Features

We explored changes in spatiotemporal gait parameters during (1) ST‐gait, (2) DT‐gait, (3) ST‐gait turns and (4) TUG after 12 weeks in the early vs. delayed start arms. The interaction effect of group (Modafinil vs. placebo) and time (0 and 12 weeks) showed no statistically significant changes in any of the 132 mean and variability parameters. However, there were several parameters nearing significance, especially in turn parameters (Table 3). For example, those on Modafinil increased their turn width 8.2 cm more than the placebo group [95% CI: (−1.1 cm, +17.5 cm); *p* = 0.081].

**TABLE 3:** Interaction effect of Group (Modafinil vs. placebo) from 0 to 12 weeks in spatiotemporal gait parameters.

	Estimate ± Standard error	df	*t*‐statistic	*p*‐value	95% CI	Covariance type
Turns						
Mean – turn width	8.2 ± 4.4	17	1.86	0.08	(−1.1, 17.5)	cs
Mean—Stance COP‐distance	−1.8 ± 1.0	17	−1.81	0.09	(−3.9, 0.3)	cs
Mean—turn steps	9.7 ± 5.6	17	1.75	0.10	(−2.0, 21.4)	csh
Mean—stance percent	4.2 ± 2.7	17	1.58	0.13	(−9.8, 1.4)	csh
CV–stance percent	−9.1 ± 5.1	17	−1.80	0.09	(−19.9, 1.6)	csh
CV–turn length	8.9 ± 5.7	17	1.56	0.14	(−3.1, 20.9)	cs
Timed‐up‐and‐go						
CV – stand‐to‐sit time	0.005 ± 0.003	18.5	1.85	0.08	(−0.001, 0.011)	csh

Abbreviations: CI, Confidence interval; CS, compound symmetry; CSH, heterogeneous compound symmetry; CV, % coefficient of variability.

#### 3.3.2 Changes in Freezing of Gait Episodes

**FIGURE 3:**
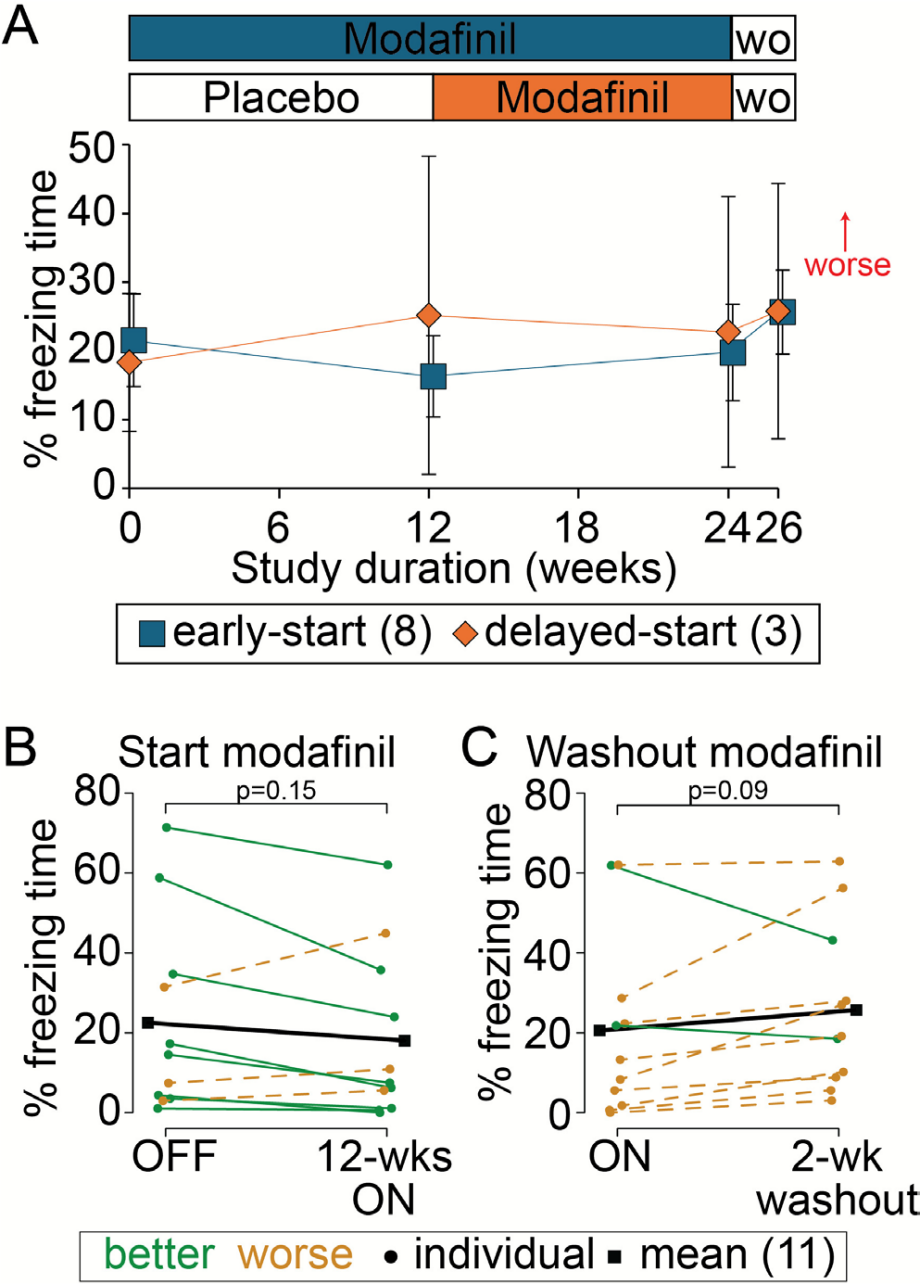
Post hoc video‐based freezing episode identification. (A) The percent freezing time (%FT) and standard deviations are plotted for the 8 early‐start (blue squares) and 3 delayed‐start (orange diamonds) participants who completed all 6 study visits and had quantifiable freezing of gait on the single‐task gait assessment at their initial visit. Collapsing the early‐ and delayed‐start groups displayed in (A), individual trajectories of %FT are plotted when transitioning (B) from OFF‐modafinil to 12 weeks of 50 mg modafinil, and (C) from ON‐modafinil to no modafinil for 2 weeks (the washout period).

There was high within subject variability in FT and %FT with no clear difference in the early‐ vs. delayed‐start groups (Table S3). As there were drop‐outs during the course of the study, and not all participants had quantifiable witnessed freezing episodes during their initial gait assessments, we selected participants who completed all study visits and had witnessed freezing during the ST‐gait trial at baseline (3 early‐start and 2 delayed‐start participants were thereby excluded). While there was no statistically significant difference in freezing measures at any time point, the expected trends for a delayed start trial type were observed (Figure 3). Comparing weeks 0 and 12, as hypothesized, the %FT decreased in the early‐start participants and increased in the delayed‐start participants (Figure 3A). Furthermore, after starting modafinil at week 12, %FT started to decrease in the delayed‐start participants. After stopping modafinil for 2 weeks at week 24, %FT increased for both groups by week 26 (Figure 3A).

As the study was blinded, we also collapsed the groups to study the effect of modafinil. After 12 weeks of modafinil, %FT decreased in 8 of the 11 participants (Figure 3B, solid green lines), on average by 4% (Figure 3B, solid blackline). Similarly, after 2 weeks of modafinil washout, %FT increased in 9 of the 11 participants (Figure 3C, dashed orange lines), on average by 5% (Figure 3C, solid blackline).

## 4 Discussion

The primary and secondary endpoints of this study did not meet their hypothesized results. However, results suggest that larger studies of modafinil are indicated. Even in a small sub‐cohort of 11 people selected because they had quantifiable FOG at their initial visit, appropriate trends were noted. In about three‐quarters of these participants there was improvement in percent freezing time (8/11) after 12 weeks of modafinil treatment, and subsequent worsening after 2 weeks of modafinil washout (9/11; Figure 3). As there are currently limited pharmacological treatments for this debilitating gait disorder outside of levodopa, further evaluation of modafinil appears to be reasonable. There are also other important lessons to be learned from this clinical trial so that the field can move forward in developing therapeutics for FOG, and better clinical trials in the future [55, 56].

After this trial was undertaken, other work confirmed that the FOG‐Q is not a good outcome measure for clinical trials [57]. There are several reasons for this. First, the FOG scales are based on historical reports from PwPD on the severity of their freezing, and since cognition in people with FOG is more affected [47, 58, 59], recall bias is a significant issue. Second, the scales are ordinal, which limits the ability to determine changes over time. For example, on the N‐FOGQ [60], people with improved FT from 30s to 12s, a 60% improvement, would be given the same score of 3 on item 4. PwPD also have difficulty judging the duration of freezing episodes and the episodes' impact on their daily life. Even a few seconds of freezing may feel like an eternity to some people, possibly depending upon their emotional state. While some people tend to discount their symptoms, reporting less impact of freezing on their quality of life (N‐FOGQ Part 3), others with low disease burden on clinical exam sometimes report higher disease impact on quality of life scales, adding to the heterogeneity of these results. There are also times in the clinic when freezing is clearly witnessed on examination, but not reported by PwPD or their caregivers.

Therefore, quantification of FT from freezing episodes is suggested as a more reliable outcome measure and has been used in several recent studies [52, 61]. We therefore analyzed our video recordings using this time‐consuming technique. Even in a relatively small number of 11 participants who completed the study and had quantifiable visualized freezing at the initial visit, the reduction in %FT after starting modafinil and the worsening in %FT after washing out modafinil for 2 weeks was nearing significance (Figure 3). Furthermore, the concordance in direction of change: improvement with drug and worsening after washout was seen in over 70% of these participants, supporting the potential therapeutic benefit of modafinil. With just 50 participants in a future cross‐over study, there would be enough power to show modafinil reduces %FT by at least 0.5 SD (assuming a modest within‐individual correlation of 0.33). We consider a 0.5 SD improvement in %FT to be a clinically meaningful improvement.

Video‐based freezing episode identification is not without issues. The process is time consuming and requires training of study team members to accurately identify freezing episodes; programs for which are under development by the International Consortium for Freezing of Gait (ICFOG) [62]. To the untrained eye, differentiating volitional pauses in walking from freezing can be difficult, say if a participant stops to make a comment or take a break after a long freezing episode. Audio can be useful to differentiate these cases. To analyze just the ST‐gait freezing episodes took a movement disorders specialist (TV) several months to accurately identify the onset and end of freezing episodes. As previously reported, and evident in our study, if people do not freeze in the lab setting variability increases, thereby lowering the chance of a favorable study outcome [5].

Another challenge is that mild FOG can be difficult to elicit in the lab setting, as it improves under focused attention during gait evaluations. While dual‐task distraction tasks and repetitive 360° turning can help provoke freezing in the lab [5, 63, 64], the latter is not routinely performed in daily life and may not relate to everyday freezing. Freezing is more common at home, where specific spatial contexts—narrow corridors, kitchen spaces, tightly spaced furniture—may commonly provoke freezing [5]. No such triggers existed in our study environment. While video‐based quantification can provide accurate characterization of short time durations (minutes), moving to the real‐world quantification of freezing will require assimilation of much longer durations (hours to days) where the laborious video‐based process will just not be feasible. The use of automated freeze detection strategies [65, 66, 67], from wearable sensors, pressure insoles, or devices placed in the environment could overcome this limitation, but these strategies must be validated against the current gold standard of video‐based visual freeze detection by experts.

Defining the minimal clinically meaningful change for objective measures is critical. Quantitative measures will be more sensitive to within‐individual change (or differences between populations) and will be more widely interpretable than our subjective, ordinal measures. However, if these objective changes are not translated into, or used to define clinical outcomes, then the lack of precision and accuracy in currently used subjective outcomes will keep our field from advancing. With increasing emphasis on patient‐centered outcome measures by the FDA, defining clinical benefit will be a difficult topic to tackle and will need critical validation of any new objective outcome measure. For example, in our study, one participant demonstrated a 34% improvement (56%–37%) in %FT, a 56% worsening in PDQ‐39 quality of life scale (43 to 67 score), and improvement of 12% (43 to 38) and 14% (72 to 62) on the motor and total UPDRS score, respectively. Despite an improvement in objective measures of disease, including freezing episodes, subjective quality of life declined. For comparison, in the hallmark ELLDOPA study which established levodopa as the gold standard treatment for motor symptoms of Parkinson's disease [68], participants had a 6–9 point reduction in total UDPRS scores compared to placebo, while our example participant had a 10 point reduction. Which of these outcome measures provides a more clinically meaningful result will be an important future discussion.

When planning future clinical trials, it will also be important to consider the rate of disease progression in the population of PwPD with FOG. We previously showed that continuous‐gait in PwPD with FOG declines at a faster rate compared to PwPD without FOG [48]. We subsequently showed that people who convert to a freezer phenotype over time show a decline in continuous‐gait, at a rate and spatiotemporal pattern, more similar to those who already had FOG than those who did not [69]. This rate of decline in objectively measured continuous‐gait parameters also better predicted future conversion from a non‐freezer to a freezer than cross‐sectional parameters at a single time point [69]. While limited by small numbers, Figure 3A demonstrates similar trends in the progression of freezing severity over the study duration. In the delayed‐start group, %FT was increasing on placebo from week 0–12, while in the early‐start group, after initial improvement on modafinil from week 0–12, %FT was also increasing from week 12–24 while continuing modafinil. After washout at week 26, %FT was on average 5% higher in both groups than at baseline. The possibility that the efficacy of modafinil wears off over time or the findings are random chance cannot be excluded. We suggest however that the progression of gait and freezing severity, even in a short 6‐month trial, should be considered when designing future clinical trials.

Currently we know that the incidence of FOG increases over the disease course, occurring in around 7% of people with early PD [3], 50% mid‐way through disease [11], and in up to 92% by the time of death [70]. Accurate identification of individuals likely to develop FOG or remain non‐freezers over a trial period is essential to ensure appropriate enrollment and minimize false‐negative outcomes in future disease‐modifying studies. Larger populations of PwPD without FOG, prospectively monitored with multimodal evaluations longitudinally, are needed to develop prediction algorithms [48, 69, 71, 72]. Future collaborative efforts to combine available datasets [73] will also be helpful.

In summary, while we found little evidence in support of our hypothesis on primary and secondary outcome measures, post hoc results provide supportive evidence for pursuing a larger study of modafinil for the treatment of FOG. Our study also provides important lessons for the design of symptomatic and neuroprotective trials in the future.

## Author Contributions

Tuhin Virmani was involved in conception and design of the study, acquisition and analysis of data and drafting of the manuscript and figures, and editing and approval of the submitted manuscript. Lakshmi Pillai was involved in acquisition and analysis of the data and drafting sections of the manuscript and editing and approval of the submitted manuscript. Reid D. Landes was involved in the design of the study, analysis of the data and drafting a significant portion of the manuscript and figures and editing and approval of the submitted manuscript. Aliyah Glover and Shannon Doerhoff were involved in acquisition of the data and editing and approval of the submitted manuscript. Jennifer Kleiner was involved in design of the study and editing and approval of the submitted manuscript. Mitesh Lotia was involved in design of the study, monitoring safety during the trial, and editing and approval of the submitted manuscript. Rohit Dhall was involved in design of the study, acquisition of data and editing and approval of the submitted manuscript. Edgar Garcia‐Rill was involved in conception and design of the study and editing and approval of the submitted manuscript.

## Funding

This work was supported by the University of Arkansas for Medical Sciences. Parkinson's Foundation (PF‐JFA‐1935). National Institute of General Medical Sciences (GM110702).

## Conflicts of Interest

Tuhin Virmani has no relevant financial disclosures. He received research and salary support for this project from NIH/NIGMS (GM110702, PI Garcia‐Rill), Parkinson's Foundation (PF‐JFA‐1935), and the University of Arkansas for Medical Sciences Clinician Scientist program. Lakshmi Pillai has no relevant financial disclosures. She received salary support for this project from the Parkinson's Foundation (PF‐JFA‐1935, PI Virmani) and the University of Arkansas for Medical Sciences Clinician Scientist program grants to T.V., Reid D. Landes has no relevant financial disclosures. He received salary support for this project from the Parkinson's Foundation (PF‐JFA‐1935, PI Virmani) and NIH/NCATS (UL1 TR003107). Aliyah Glover has no relevant financial disclosures. She received salary support for this project from the NIH/NIGMS (GM110702, PI Garcia‐Rill), Parkinson's Foundation (PF‐JFA‐1935, PI Virmani), and the University of Arkansas Clinician Scientist for Medical Sciences program grants to T.V., Shannon Doerhoff has no relevant financial disclosures. She received salary support from the University of Arkansas for Medical Sciences. Jennifer Kleiner has no relevant financial disclosures. She received salary support from the University of Arkansas for Medical Sciences. Mitesh Lotia has no relevant financial disclosures. He received salary support from the University of Arkansas for Medical Sciences. Rohit Dhall has no financial disclosures related to this study. He received support as an investigator for Amneal, Rho Inc., Lundbeck, Amylyx, UCB Pharma, Northeast ALS Consortium, AbbVie, Neurocrine, Neuroderm, Biohaven, Sage, Inhibikase, Takeda, and consultant fees from AbbVie and Best Doctors. He also received salary support from the University of Arkansas for Medical Sciences. Edgar Garcia‐Rill has no relevant financial disclosures. He received salary support from the NIH/NIGMS (GM110702, PI Garcia‐Rill) as part of this project.

## Supporting information


**Data S1:** Supplementary Information.

**Table S1:** Spatiotemporal gait and turn parameters—sources and definitions.
**Table S2:** All adverse events reported during the first 12 weeks in participants completing at least one post‐randomization visit.
**Table S3:** Quantified Total and Percent Freeze Time of all participants who completed Visit 3.

## Data Availability

The data that support the findings of this study will be made openly available in figshare with an associated DOI, once the manuscript is accepted for publication.
