# Supplementary material for: Lessons Learned From a Delayed‐Start Trial of Modafinil for Freezing of Gait in Parkinson's Disease
Source: Ann Clin Transl Neurol. 2025 Dec 8;13(5):899–910. doi: 10.1002/acn3.70276 (PMC13161890; doi:10.1002/acn3.70276)
Supplement: Supplementary file 1 — Data S1: Supplementary Information. [file ACN3-13-899-s002.docx]

**Study Title:** Modafinil as a novel therapy for the treatment of freezing of gait in Parkinson’s disease

**Principal Investigator:** Tuhin Virmani, MD, PhD

University of Arkansas for Medical Sciences

4301 W. Markham Street, Slot # 500

Little Rock, AR 72205

Telephone: *501-686-7235*

*Fax: 501-686-8689*

Email: *TVirmani@uams.edu*

**Sub-Investigator(s):** Edgar Garcia-Rill, PhD

Professor and Director

Center for Translational Neuroscience

Department of Neurobiology and Developmental Sciences

Rohit Dhall, MD

Associate Professor

Department of Neurology

Jennifer Kleiner-Fausett, PhD

Associate Professor

Department of Psychiatry

**Biostatistician:** David Williams, PhD

University of Arkansas for Medical Sciences

4301 W. Markham Street, Slot # 781

Little Rock, AR 72205

Telephone: *501-526-6721*

Email: *WillamsDavidK@uams.edu*

**Study Coordinator:** Shannon Doerhoff, APRN

Department of Neurology

**Medical Monitor:** Mitesh Lotia, MD

Assistant Professor

Department of Neurology

**Study location(s):** University of Arkansas for Medical Sciences, 5^th^ floor JTS.

**Sponsor:** UAMS

**Funding Source**: UAMS Clinician Scientist Program

CTN RFA (active May 1, 2017)

**Version No.:** 4

**Version Date:** 06/28/17

TABLE OF CONTENTS

[1.0 LIST OF ABBREVIATIONS 5](#_Toc476665646)

[2.0 STUDY SUMMARY 6](#_Toc476665647)

[3.0 BACKGROUND AND RATIONALE 7](#_Toc476665648)

[3.1 Disease Background 7](#_Toc476665649)

[3.2 Investigational Product Background 7](#_Toc476665650)

[3.3 Rationale 9](#_Toc476665651)

[4.0 STUDY OBJECTIVES 9](#_Toc476665652)

[4.1 Primary Objectives 9](#_Toc476665653)

[4.2 Secondary Objectives 9](#_Toc476665654)

[4.3 Exploratory Objectives 10](#_Toc476665655)

[4.4 Endpoints 10](#_Toc476665656)

[5.0 STUDY POPULATION 11](#_Toc476665657)

[5.1 Inclusion Criteria 11](#_Toc476665658)

[5.2 Exclusion Criteria 11](#_Toc476665659)

[5.3 Accrual Goal 12](#_Toc476665660)

[5.4 Recruitment Plan 12](#_Toc476665661)

[6.0 INVESTIGATIONAL PRODUCT 12](#_Toc476665662)

[6.1 Test Article 12](#_Toc476665663)

[6.2 Treatment Dosage and Administration 13](#_Toc476665664)

[6.3 Toxicities and Dosing Delays/Dose Modifications 13](#_Toc476665665)

[6.4 Concomitant Medications/Treatments 14](#_Toc476665666)

[6.5 Other Modalities or Procedures 14](#_Toc476665667)

[6.6 Duration of Therapy 14](#_Toc476665668)

[6.7 Duration of Follow Up 14](#_Toc476665669)

[6.8 Removal of Patients from Protocol Therapy 14](#_Toc476665670)

[6.9 Patient Replacement 14](#_Toc476665671)

[7.0 STUDY PROCEDURES 14](#_Toc476665672)

[7.1 Screening/Baseline Procedures 14](#_Toc476665673)

[7.2 Procedures During Treatment 15](#_Toc476665674)

[7.3 Schedule of Time and Events Table 17](#_Toc476665675)

[7.4 Removal of Subjects from Study 18](#_Toc476665676)

[8.0 Outcome Measures 18](#_Toc476665677)

[8.1 Improvement in FOG 18](#_Toc476665678)

[9.0 ADVERSE EVENTS 20](#_Toc476665679)

[9.1 Experimental Therapy 20](#_Toc476665680)

[9.2 Adverse Event Monitoring 22](#_Toc476665681)

[9.3 Definitions 23](#_Toc476665682)

[9.4 Steps to Determine If an Adverse Event Requires Expedited Reporting 24](#_Toc476665683)

[9.5 Reporting Requirements for Adverse Events 24](#_Toc476665684)

[9.6 Unblinding Procedures 25](#_Toc476665685)

[9.7 Stopping Rules 25](#_Toc476665686)

[10.0 STATISTICAL CONSIDERATIONS 25](#_Toc476665687)

[11.0 STUDY MANAGEMENT 25](#_Toc476665688)

[11.1 Institutional Review Board (IRB) Approval and Consent 26](#_Toc476665689)

[11.2 Data Management and Monitoring/Auditing 26](#_Toc476665690)

[11.3 Adherence to the Protocol 26](#_Toc476665691)

[11.4 Amendments to the Protocol 26](#_Toc476665692)

[11.5 Record Retention 26](#_Toc476665693)

[11.6 Dissemination of data 27](#_Toc476665694)

[11.7 Obligations of Investigators 27](#_Toc476665695)

[12.0 REFERENCES 27](#_Toc476665696)

# LIST OF ABBREVIATIONS

AE: Adverse Event

AES-S: Apathy evaluation scale

CBGD: Corticobasal ganglionic degeneration

CBS: Corticobasal Syndrome

DBS: Deep Brain Stimulators

ET: Essential tremor

FAB: Frontal Assessment Battery

FOG: Freezing of gait

FOG-Q: Freezing of gait questionnaire

MoCA: Montreal cognitive assessment

MSA: Multiple systems atrophy

PAGF: Primary akinesia with gait freezing

PD: Parkinson’s disease

PDQ-39: Parkinson’s disease questionnaire for quality of life

PKMAS: Protokinetic movement analysis software

PPFG: Primary progressive freezing of gait

PPN: Pedunculopontine nucleus

PSP: Progressive supranuclear palsy

RBD: Rapid eye movement sleep behavior disorder

SCOPA-COG: Scales for outcome in Parkinson’s disease – cognition

SSRIs: selective serotonin reuptake inhibitors
SWS: stand-walk-sit

TCAs: tricyclic antidepressants

UPDRS: Unified Parkinson’s Disease Rating Scale

# STUDY SUMMARY

| Title | Modafinil as a novel therapy for the treatment of freezing of gait in Parkinson’s disease |
| --- | --- |
| Short Title | Modafinil for FOG |
| Protocol Number | MODFOG01 |
| Phase | Pilot, feasibility |
| Methodology | Randomized, placebo-controlled delayed start |
| Study Duration | 1 year |
| Study Center(s) | Single-center |
| Objectives | Safety and efficacy of Modafinil for the treatment of freezing of gait in Parkinson’s disease |
| Number of Subjects | Enrolling 25 for a total of 20 |
| Diagnosis and Main Inclusion Criteria | Idiopathic Parkinson disease with freezing of gait on stable Parkinson disease treatment for 3 months. |
| Study Product(s), Dose, Route, Regimen | Modafinil 50 mg capsule oral daily, Placebo capsule oral daily |
| Duration of administration | 24 weeks (early-start), 12 weeks (delayed-start) |
| Reference therapy | none |
| Statistical Methodology | The study is a prospective repeated measures longitudinal design. A primary statistical goal is to estimate the mean response at all six time points for each group and subsequently estimate changes in the outcome measures for each study group from baseline. |

# BACKGROUND AND RATIONALE

## Disease Background

Patients with Parkinson Disease (PD) typically present with bradykinesia, rigidity, and frequently resting tremor along with other hallmarks such as shuffling gait, decreased blinking, decreased arm swing, hypophonia, hypomimia, micrographia, and decreased smell and taste. In the later stages of the disease, dementia, postural instability, and freezing of gait are also common. Freezing of gait (FOG) is one of the more debilitating motor complications of PD.^1, 2^ It is manifested by the feet “sticking to the ground” for several seconds during active movement. FOG occurs initially when the patient is turning or when initiating gait (start-hesitation). Later, it occurs when the patient reaches a destination, such as a chair, and the patient stops too soon, before fully reaching the desired destination.

FOG is a complex disorder that can also be seen as an entity of its own in primary progressive freezing of gait (PPFG), primary akinesia with gait freezing (PAGF), or in atypical Parkinsonism such as vascular Parkinsonism, progressive supranuclear palsy (PSP), corticobasal syndrome (CBS), and multiple systems atrophy (MSA). FOG has been thought to be due either to a variant in the disease process in certain individuals, or potentially as a side effect of levodopa, one of the mainstay medications used to treat the symptoms of PD. Earlier onset FOG has been associated with earlier onset of dyskinesias and postural instability, the presence of hallucinations and greater neocortical Lewy Body burden.^3^ FOG in PD can occur both as a phenomenon of the “OFF state” which improves with addition of dopaminergic medications (i.e., either levodopa or dopamine agonists), but in an unknown percentage it also occurs in the “ON state” and does not respond to increased levodopa. In fact FOG in the “ON state” is often reported to worsen with incrementally higher levodopa doses.

The presence of gait freezing results in a significant worsening of quality of life for patients. Freezing leads to decreased mobility, increased rates of falls, and subsequent fear of falling, which further limits mobility.^4-6^ Morbidity associated with hip fractures from falls leads to hospitalizations, surgeries and further dependence on caregivers. Often, patients lapse into a wheelchair- or home-bound state, increasing pressure on care givers. While OFF state FOG usually responds to increases in levodopa dosing, ON state FOG does not, and can even worsen.^7^

Unfortunately, no good alternative treatments exist for FOG. Methylphenidate, a stimulant used in the treatment of Attention deficit hyperactivity disorder, inhibits presynaptic dopamine^8^ and the noradrenaline transporter. Used at high doses (1 mg/kg/day), methylphenidate has been shown to decrease the number of steps taken by PD patients who have undergone subthalamic nucleus (STN) DBS in the stand-walk-sit (SWS) test, but it has not been widely adopted due to cardiac side effects.^9^ Therefore, we will test whether another stimulant, Modafinil, could serve as a novel treatment for FOG.

## Investigational Product Background

The pedunculopontine nucleus (PPN) is a part of the reticular activating system, and arousing stimuli simultaneously activate ascending projections to the intralaminar thalamus triggering high frequency cortical activity, and descending projections to the reticulospinal systems altering posture and locomotion. This has led to the PPN being used as a target for deep brain stimulation (DBS) in the treatment of PD, and proposed as a potential target for treating FOG in PD.^10-12^ PPN neurons fire at beta/gamma frequencies during waking and REM sleep but not during slow wave sleep.^13^ These oscillations are mediated by high threshold voltage dependent N- and P/Q-type calcium channels.^14^ Garcia-Rill and colleagues have shown that beta/gamma oscillations in PPN neurons were blocked by KN-93 (a CAMKII activation inhibitor), which suggests that at least some cells produce oscillations through the CAMKII pathway.

Modafinil, a stimulant approved by the FDA for treatment of narcolepsy (decreases daytime sleepiness and regulates sleep-wake cycles), has a number of putative sites of action.^15^ Garcia-Rill and colleagues, as well as a group of researchers at NYU have both shown that modafinil works by increasing neuronal electrical coupling, and this action of modafinil is also modulated by the CAMKII pathway as KN-93 also inhibits the effects of modafinil.^16, 17^ Modafinil has also been shown to bind to the dopamine transporter and prevent dopamine reuptake, increasing the level of dopamine in the synaptic cleft.^18^

Modafinil is a 1:1 racemic compound whose enantiomers have different pharmacokinetics (The half-life of R-modafinil is approximately three times that of S-Modafinil in humans). The effective elimination half-life of Modafinil is about 15 hours with linear kinetics upon multiple dosing of 200-600 mg/day with steady state reached after 2-4 days of dosing for narcolepsy. Modafinil is absorbed orally with peak plasma concentrations in 2-4 hours. The major route of elimination is metabolism (approximately 90%) by the liver with subsequent renal elimination of metabolites. In-vitro data suggests that Modafinil is a weak inducer of CYP3A activity in a concentration dependent manner and therefore may reduce the effectiveness of drugs that are substrates for CYP3A enzymes (e.g. steroidal contraceptives, cyclosporine, midazolam, triazolam). Data also suggest that Modafinil is a weak inducer of CYP1A2, CYP2B6, and CYP2C9 (warfarin and phenytoin). Modafinil is also a reversible inhibitor of CYP2C19 and therefore may increase concentrations of drugs metabolized by this enzyme (e.g. phenytoin, diazepam, propranolol, omeprazole, and clomipramine). Modafinil may interact with some CNS active drugs that PD patients may be using including certain tricyclic antidepressants (TCAs) (metabolized by CYP2C19) and selective serotonin uptake inhibitors (SSRIs) (metabolized by CYP2D6). Also concomitant administration of quetiapine reduced the systemic exposure of quetiapine.

Based on data from the Garcia-Rill lab and a prior unpublished trial at the Little Rock VA on Modafinil in PTSD patients, subjects were found to be more jittery at doses of 100mg and higher (unpublished, personal communication). Therefore for this study low dose Modafinil at 50 mg orally daily will be trialed (the FDA approved recommended dosing for Narcolepsy is 200 mg/day for Narcolepsy). Additionally small studies in elderly populations suggest decreased clearance of Modafinil in elderly populations. One study showed approximately 20% decrease in oral clearance in 12 subjects given a single dose of 200 mg modafinil (mean age 63 years, range 53-72 years), while a study with multiple doses of 300 mg/day Modafinil in 12 subjects (mean age 82 years, range 67-87 years) showed mean plasma levels were two times those in historical younger subjects.

## Rationale

FOG is present in approximately 60% of patients with PD and is a major cause of gait impairment leading to falls, and the morbidity and decreased quality of life. Currently, other than levodopa therapy, which can be limited in later stages of disease when FOG occurs by dose dependent side effects, no alternative treatments exist. Modafinil, as described above, has been shown by the Garcia-Rill lab to modulate pathways in the PPN that link arousal and gait dynamics. Additionally, PPN DBS has also been shown to modulate gait and falls in patients. As such, we propose to study whether Modafinil can improve FOG in PD. For this pilot study, we will focus on recruiting PD patients with FOG and use a delayed start trial model to determine whether FOG improves compared to placebo. We chose the delayed start trial model as opposed to just having a placebo arm as it will likely help with recruitment and subject retention if all subjects know that at least during 3 months of the trial they will all be receiving active drug. Additionally, preliminary data (unpublished, UAMS IRB#203234) from my lab suggests that PD patients with FOG have a faster rate of decline in objective gait parameters compared to PD patients, even over a period of 3-6 months. Therefore, with the delayed start model we will also determine if there are any changes in the slope of decline in PD-FOG patients over the period of the trial.

# STUDY OBJECTIVES

This is a single-center, prospective, randomized, double-blinded, placebo-controlled, delayed-start pilot trial involving up to 25 subjects with PD and FOG. Subjects will be randomized 1:1 to early start (24 weeks active treatment) or delayed start (12 weeks placebo followed by 12 weeks active treatment) oral Modafinil 50 mg/day. Following 24 weeks of treatment, a 2 week washout of the drug will be performed with a follow-up assessment at 26 weeks.

## Primary Objectives

### To determine if 50 mg/day Modafinil decreases FOG in PD patients with FOG compared with placebo as measured by the Giladi Freezing of gait questionnaire (FOG-Q).

### To determine if 50 mg/day Modafinil decreased the decline stride length seen in PD patients with FOG.

## Secondary Objectives

### To determine if 50 mg/day Modafinil improves motor function in PD patients with FOG compared to placebo as measured by the UPDRS Part III motor subscale.

### To describe the adverse events associated with Modafinil dosed at 50 mg/day for 3-6 months in PD patients.

### To determine if quality of life is improved with Modafinil as measured by the PDQ-39.

### To determine if sleep disorders are improved with Modafinil as measured by the RBD scale and Epworth Sleepiness Scale.

## Exploratory Objectives

### To determine if Modafinil 50 mg/day improves cognitive function in PD patients with FOG compared to placebo as measured by the Montreal Cognitive assessment, Stroop test, Frontal Assessment Battery and Scales for Outcome in Parkinson disease, Cognitive assessment (SCOPA-cog)

## Endpoints

### Primary Endpoints

#### To determine if 50 mg/day Modafinil decreases freezing of gait in PD patients with FOG compared with placebo as measured by the Giladi Freezing of gait questionnaire (FOG-Q) after 12 weeks of active treatment or placebo. Subjects who drop out of the study after at least 6 weeks of either treatment arm will be included in this analysis.

#### To determine if 50 mg/day Modafinil decreased the decline stride length seen in PD patients with FOG. The rate of decline in stride length will be compared plotting the stride length at steady state in subjects in the early and late-start arms to determine if the rate of decline improves in subjects taking Modafinil in the early-start arm and whether this rate of decline also improves in the second phase of the trial in the delayed-start arm.

#### We will also determine if at the completion of 24 weeks, the early-start arm has differential change in slope of other continuous gait parameters (stride width, gait cycle time, gait cycle velocity, swing and stance percent, total single and double support time, cadence) compared to the delayed start arm suggesting that Modafinil slows the progression of the gait decline.

### Secondary Endpoints

#### To determine if 50 mg/day Modafinil improves motor function in PD patients with FOG compared to placebo as measured by the UPDRS Part III motor subscale after 12 weeks of active treatment or placebo. Subjects who drop out of the study after at least 6 weeks of either treatment arm will be included in this analysis.

#### To describe the adverse events associated with Modafinil dosed at 50 mg/day in patients treated for 3 months (delayed start arm) and 6 months (early start arm) in Parkinson’s disease patients.

#### To determine if quality of life is improved with Modafinil as measured by the PDQ-39 after 12 weeks of active treatment or placebo. Subjects who drop out of the study after at least 6 weeks of either treatment arm will be included in this analysis.

#### To determine if sleep disorders are improved with Modafinil as measured by the RBD scale and Epworth Sleepiness Scale after 12 weeks of active treatment or placebo. Subjects who drop out of the study after at least 6 weeks of either treatment arm will be included in this analysis.

### Exploratory Endpoints

#### To determine if Modafinil 50 mg/day improves cognitive function in PD patients with FOG compared to placebo as measured by the Montreal Cognitive assessment, Stroop test, Frontal Assessment Battery and Scales for Outcome in Parkinson disease, Cognitive assessment (SCOPA-cog) after 12 weeks of active treatment or placebo. Subjects who drop out of the study after at least 6 weeks of either treatment arm will be included in this analysis. Additionally we will determine if at the end of 24 weeks, whether the early-start group had additional benefit of the additional 12 weeks of Modafinil treatment.

# STUDY POPULATION

Eligibility waivers are not permitted. Subjects must meet all of the inclusion and exclusion criteria to be enrolled in the study. Study treatment may not begin until a subject is enrolled.

## Inclusion Criteria

- Age ≥ 50 years.
- Diagnosis of idiopathic PD on UK brain bank criteria.
- Presence of FOG based on objective assessment by the movement disorders neurologist.
- FOG-Q score > 8.
- Stable PD therapy (including medications and stimulation) for a period of 3 months prior to trial enrollment.
- Women of child-bearing potential and men must agree to use adequate contraception (hormonal or barrier method of birth control; abstinence) prior to study entry, for the duration of study participation, and for 90 days following completion of therapy. Should a woman become pregnant or suspect she is pregnant while participating in this study, she should inform her treating physician immediately.
  - A female of child-bearing potential is any woman (regardless of sexual orientation, having undergone a tubal ligation, or remaining celibate by choice) who meets the following criteria:
    - Has not undergone a hysterectomy or bilateral oophorectomy; or
    - Has not been naturally postmenopausal for at least 12 consecutive months (i.e., has had menses at any time in the preceding 12 consecutive months).
- Ability to understand and the willingness to sign a written informed consent.

## Exclusion Criteria

- Patients on antidopaminergic medications for a period of less than 1 year from date of enrollment.
- Patients who may require adjustment of their PD medications over the 6 month period of the trial.
- History of allergic reactions to Modafinil or armodafinil.
- Uncontrolled intercurrent illness including, but not limited to, ongoing or active infection, symptomatic congestive heart failure, unstable angina pectoris, cardiac arrhythmia, mitral valve prolapse, left ventricular hypertrophy, chronic obstructive pulmonary disease, known malabsorption syndromes, renal disease or hepatic disease, or psychiatric illness/social situations that would limit compliance with study requirements.
- Individuals who are pregnant or breastfeeding
- Non-english speaking individuals who are unable to complete the questionnaires and other assessments in English and/or follow instructions in English.

## Accrual Goal

The goal for this study is to complete 20 subjects with PD and FOG, randomized 1:1 to early start and delayed start with Modafinil. Up to 25 patients may be enrolled in order to accomplish the goal.

## Recruitment Plan

Subjects will be recruited from the population of patients already performing routine gait assessments as part of an ongoing IRB by the PI (Dr. Virmani; UAMS IRB # 203234). In addition subjects will be recruited from the PI’s clinic patient population and from the two other movement disorders neurologists at UAMS (Dr. Lotia and Dr. Dhall).

# INVESTIGATIONAL PRODUCT

## Test Article

**Modafinil**

For this study Modafinil 50 mg oral capsules will be produced and dispensed by the UAMS pharmacy in gelatin capsules with microcrystalline cellulose as filler. Subjects will take Modafinil 50 mg capsule oral daily for either 12 weeks or 24 weeks depending upon whether they are in the early-start or delayed-start arm of the trial as described below (see Figure 1).

**Placebo**

For this study placebo capsules will be made of gelatin capsules filled with microcrystalline cellulose and will be identical to the Modafinil capsule. Subjects in the delayed start arm will receive 12 weeks of placebo followed by 12 weeks of Modafinil 50 mg oral daily (see Figure 1)

### Dispensing

Modafinil and placebo capsules will be produced and dispensed by the pharmacy in labeled packets with appropriate instructions on how they should be taken. Both will be dispensed by the UAMS pharmacy in 6 week increments at study visits 1-4.

### Treatment Compliance

### A pill diary will be provided to the patients in order to try and ensure compliance with the treatment regimen. Patients will also be asked to return the pill containers at each visit to help ensure compliance. If patients are unable to provide the pill diary then they will be asked to sign a form stating their compliance with the study dosing regimen.

## Treatment Dosage and Administration

The research pharmacy will create a randomization log, randomizing 20 subjects 1:1 to early start or delayed start modafinil (Figure 1). Subjects will be assigned the next line in the log in order of treatment. Subjects in the early-start arm will receive Modafinil 50 mg oral daily (a.m.) for 24 weeks while subjects in the delayed-start arm will receive oral placebo for the first 12 weeks of active treatment followed by Modafinil 50 mg oral daily for weeks 13-24 of the active treatment period. The drug will be washed out for a 2 week period with repeat assessments to determine any withdrawal symptoms.


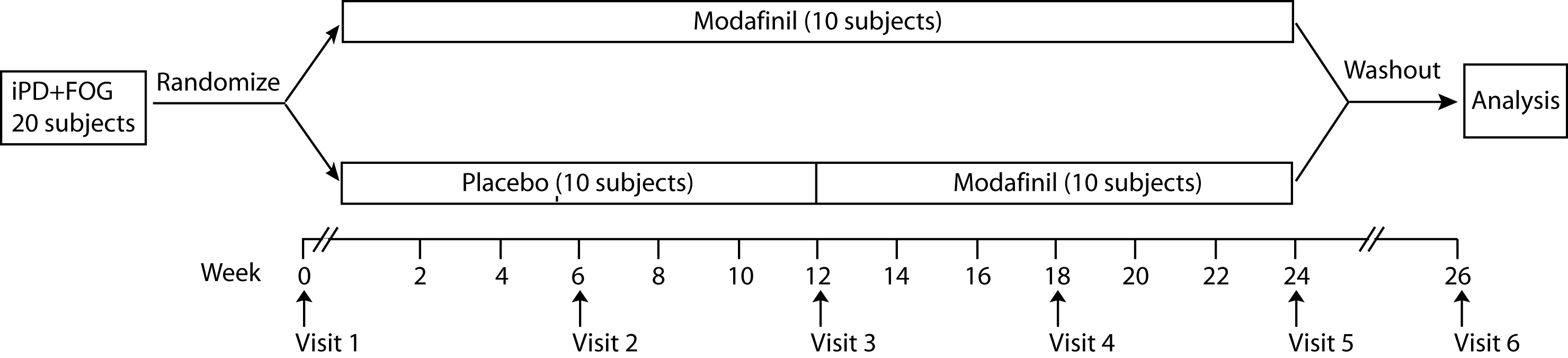


Figure 1: Delayed-start trial model with 1:1 randomization into an early-start arm with 10 subjects receiving Modafinil 50 mg oral daily for 24 weeks and 10 subjects randomized to a delayed-start arm receiving placebo for the 1^st^ 12 weeks followed by Modafinil 50mg oral daily for weeks 13-24. Visits will be performed at initiation and randomization, followed by 6, 12, 18 and 24 week visits in the active phase with the final visit performed after a 2 week drug washout phase.

## Toxicities and Dosing Delays/Dose Modifications

Any patient who receives treatment on this protocol will be evaluable for toxicity. Each patient will be assessed for the development of toxicity as described in section 9. If patients develop the following toxic side effects to Modafinil 50 mg (current FDA dosing for narcolepsy is 4 times this dose at 200 mg), they will be withdrawn from the study.

### Stevens-Johnson Syndrome

### Angioedema and Anaphylaxis

### multi-organ failure

### persistent excessive daytime sleepiness

### psychiatric symptoms (any out of mania, delusions, suicidal ideations, paranoid delusions, or auditory hallucinations)

### cardiovascular events (chest pain, palpitations, dyspnea, ischemic T-wave changes on electrocardiogram)

Missed doses will be tabulated using a pill diary and if more than 14 days of dosing was missed in either the first 12 weeks or the second 12 weeks of the active treatment phase then this would warrant removal of the patient from the study.

## Concomitant Medications/Treatments

All antidopaminergic agents (including typical and atypical antipsychotics, antidopaminergic antiemetics and antidopaminergic antidepressants) are restricted during the period of this trial and would in any case be contraindicated for use in patients with Parkinson disease.

## Other Modalities or Procedures

No other modalities (e.g., surgery, radiotherapy) or procedures (e.g., hematopoietic stem cell transplantation) will be used in the protocol treatment.

## Duration of Therapy

In the absence of treatment delays due to adverse events, treatment may continue for a total of 24 weeks or until:

- Disease progression is documented per the judgment of the investigator
- Inter-current illness that prevents further administration of treatment
- Unacceptable adverse event(s) (see section 6.3)
- Patient decides to withdraw from the study, **OR**
- General or specific changes in the patient’s condition render the patient unacceptable for further treatment in the judgment of the investigator.

## Duration of Follow Up

Patients will be reassessed at a 2 week follow-up visit after completion of the active treatment arm to determine if there are any adverse events associated with withdrawal of Modafinil 50 mg. As subjects will be recruited from the population of patients already cared for at the UAMS movement disorders clinic they may continue to get regular clinic visits at UAMS for the treatment of their Parkinson disease as determined by the movement disorders neurologist after the study period has elapsed.

## Removal of Patients from Protocol Therapy

Patients will be removed from therapy when any of the criteria listed in Section 7.4 apply. Notify the Principal Investigator, and document the reason for study removal and the date the patient was removed in the Case Report Form. The patient should be followed-up per protocol.

## Patient Replacement

Subjects who have completed less than 2 weeks of the active treatment arm may be replaced with an alternative subject who would be enrolled in the same treatment arm as the subject being replaced, i.e. either the early-start or delayed-start arm. Up to 25 subjects may be recruited in order to try and obtain 20 subjects completing the study.

# STUDY PROCEDURES

## Screening/Baseline Procedures

Assessments performed exclusively to determine eligibility for this study will be done only after obtaining informed consent. Assessments performed for clinical indications (not exclusively to determine study eligibility) may be used for baseline values even if the studies were done before informed consent was obtained.

All screening procedures must be performed within 14 days prior to registration unless otherwise stated. The screening procedures include:

### *Informed Consent and HIPAA Authorization*

### *Medical history*

Complete medical and surgical history, and history of infections. Medical chart will be reviewed for potential contraindications to participation including available hepatic function tests, renal functions tests, and ECGs or echocardiograms if available.

### *Demographics*

Age, gender, race, ethnicity

### *Review subject eligibility criteria*

### *Review previous and concomitant medications*

### *Physical exam including vital signs, height and weight*

Vital signs (temperature, pulse, respirations, orthostatic blood pressure), height, weight

### *Adverse event assessment*

Baseline adverse events will be assessed. See section 9 for Adverse Event monitoring and reporting.

## Procedures During Treatment

Gait and Balance Assessments:

Subjects will be asked to walk on the 20’ long by 4’ wide pressure sensor-impregnated Zeno Walkway using a number of protocols as outlined below. These assessments will be formed at enrollment, 6 weeks on Modafinil, 12 weeks on Modafinil and 2 weeks after stopping Modafinil. See Table 1 below for detailed study schedule.

1. Walking at a comfortable pace on and off the mat, 8 lengths of the mat.
2. Walking at a comfortable pace on and off the mat, 8 lengths of the mat, with two dual-task paradigms
   1. Texting on a cellular phone
   2. Counting backwards aloud from 250
3. Walking at a comfortable pace making pivot turns on the mat, 8 lengths of the mat.
4. Walking at a comfortable pace making U-turns turns on the mat, 8 lengths of the mat.
5. Sit-Stand-Walk test: starting in a sitting position, standing up, walking to the end of the mat, pivot turn, walk back and sit back down, 4 repetition.
6. Tandem walk the length of the mat
7. Postural sway: stand with feet together for 30 seconds and then with feet in tandem for 30 seconds
8. Pull test: stand with examiner behind, pulling backwards at the shoulders at 4 different strengths twice.
   1. First time asking the subject to step back only if needed
   2. Second time asking the subject to step back every time they get pulled

Data from the Zeno Walkway will be collected using a desktop computer running the PKMAS software. The resulting data will be analyzed for standard characteristics of gait such as step time and length, stride time and length, stride velocity and acceleration, swing time, gait cycle time, center of pressure of each foot, center of body mass and coefficients of variation between these. In addition, parameters of the episodic freezing episodes such as time to overcome the freeze, center of mass variation during the freeze and amplitude and frequency of tremor in the legs during the freezing episodes will also be captured and evaluated. The program also provides raw data of every sensor reading during footfall allowing for a large number of additional parameters to be calculated and based on initial results, these parameters may be assessed in the future. The sensor data is time locked to two cameras to allow for accurate characterization of foot falls. Subjects will be assessed in the OFF levodopa state and 60 minutes after taking their regularly prescribed levodopa dose at each time point.

Additional assessments (See Table 1 below for detailed study schedule):

The following measures/scales will be performed at every visit (unless otherwise specified) by Dr. Virmani (utilizing his protected clinical research time), or other study personnel:

1. Demographic information will be recorded at screening/first visit (5 minutes).
2. Complete neurological examination will be given every 12 weeks and at visit six (15 minutes).
3. Vital Signs (orthostatic blood pressure and heart rate, weight) (5 minutes)
4. Medication profile will be recorded or reviewed every visit (5 minutes).
5. Complete movement disorders examination at every visit (10 minutes).
6. Unified Parkinson’s Disease Rating Scale (UPDRS) (10 minutes).
7. Freezing of gait questionnaire (FOG-Q) (or NFOG-Q) (2-3 minutes).
8. Schwab and England Activities of Daily Living scale (1 minute).
9. Hoehn and Yahr Scale Staging of Parkinson’s disease (no additional patient time).
10. Epworth sleepiness scale (2-3 minutes).
11. REM Sleep Behavior Disorder Screening Questionnaire (2-3 minutes).
12. Apathy Evaluation scale (AES-S) (5 minutes).
13. Parkinson’s disease questionnaire for quality of life (PDQ-39) (7-10 minutes).
14. Montreal Cognitive Assessment (MoCA) every 12 weeks and at visit six (10-15 minutes).
15. Frontal assessment battery (FAB) every 12 weeks and at visit six (5-10 minutes).
16. Stroop test every 12 weeks and at visit six (3-5 minutes).
17. Scales for outcome in Parkinson’s disease – cognition (SCOPA-COG) every 12 weeks and at visit six (10-15 minutes).

## Schedule of Time and Events Table

| **Activity** | **Visit 1 (Screening Visit)** | **Visit 2**  **(6 weeks)** | **Visit 3**  **(12 weeks)** | **Visit 4**  **(18 weeks)** | **Visit 5**  **(24 weeks)** | **Visit 6**  **(26 weeks)** |
| --- | --- | --- | --- | --- | --- | --- |
| Inclusion/Exclusion | X |  |  |  |  |  |
| Informed Consent Signed | X |  |  |  |  |  |
| HIPAA Authorization Signed | X |  |  |  |  |  |
| Demographic Information | X |  |  |  |  |  |
| Vital Signs^1^ | X | X | X | X | X | X |
| Medication Profile^1^ | X | X | X | X | X | X |
| Complete Neurological Examination^1^ | X |  | X |  | X | X |
| Complete Movement Disorders Examination^1^ | X | X | X | X | X | X |
| Gait and Balance Measurements^1^ | X | X | X | X | X | X |
| Unified Parkinson’s Disease Rating Scale (UPDRS) ^1^ | X | X | X | X | X | X |
| Freezing of Gait Questionnaire (FOG-Q or NFOG-Q) ^1^ | X | X | X | X | X | X |
| Schwab & England Activities of Daily Living Scale^1^ | X | X | X | X | X | X |
| Hoehn & Yahr Scale Staging of Parkinson’s Disease^1^ | X | X | X | X | X | X |
| Montreal Cognitive Assessment (MoCA) ^1^ | X |  | X |  | X | X |
| Frontal Assessment Battery (FAB)^1^ | X |  | X |  | X | X |
| Stroop Test^1^ | X |  | X |  | X | X |
| Scales for Outcome in Parkinson’s Disease – Cognition (SCOPA-COG) ^1^ | X |  | X |  | X | X |
| Epworth Sleepiness Scale^2^ | X | X | X | X | X | X |
| REM Sleep Behavior Disorder Screening Questionnaire^2^ | X | X | X | X | X | X |
| Apathy Evaluation Scale (AES-S)^2^ | X | X | X | X | X | X |
| Parkinson’s Disease Questionnaire for Quality of Life (PDQ-39)^2^ | X | X | X | X | X | X |

^1^ Study staff performed

^2^ Self-assessment questionnaires

## Removal of Subjects from Study

Patients can be taken off the study treatment and/or study at any time at their own request, or they may be withdrawn at the discretion of the investigator for safety, behavioral or administrative reasons. The reason(s) for discontinuation will be documented and may include:

- - - Patient voluntarily withdraws from treatment (follow-up permitted);
    - Patient withdraws consent (termination of treatment and follow-up);
    - Patient is unable to comply with protocol requirements;
    - Patient demonstrates disease progression (unless continued treatment with study drug is deemed appropriate at the discretion of the investigator);
    - Patient experiences toxicity that makes continuation in the protocol unsafe;
    - Treating physician judges continuation on the study would not be in the patient’s best interest;
    - Patient becomes pregnant (pregnancy to be reported along same timelines as a serious adverse event);
    - Lost to follow-up. If a research subject cannot be contacted despite repeated attempts during the 6 month period they will be considered lost to follow-up. All attempts to contact the subject during the 6 months must be documented and approved by the Data Monitoring Committee.

# Outcome Measures

## Improvement in FOG

### Motor assessments

Giladi freezing of gait questionnaire (FOG-Q): Validated questionnaire with 6 items rated 0-4 for a total of 24 points in total. We will be looking for a 10% change in this questionnaire to suggest a subjective improvement in freezing of gait over the course of the study.

UPDRS Part III motor score: Validated rating scale used to quantify the movement disorders motor examination including items for bradykinesia, tremor, rigidity and gait.

Gait and balance measures: Will evaluate for changes in steady state gait and balance measures using the assessments outlined in section 7.2 including mean and percent coefficient of variability in:

- Stride length
- Stride width
- Stride velocity
- Step time
- Step length
- Gait cycle time
- Swing cycle percent
- Stance cycle percent
- Single support percent
- Total double support percent
- Velocity
- Cadence
- Ambulation time

### Cognitive assessments:

The following cognitive assessments will be administered by Dr. Virmani or other trained study personnel. Dr. Kleiner-Fausett (licensed psychologist) will supervise the interpretation of these results.

Montreal cognitive assessment (MoCA): commonly used scale to screen for cognitive dysfunction in Parkinson disease patients.

Frontal assessment battery (FAB): commonly used scale to screen for frontal lobe dysfunction that can be suggestive of atypical parkinsonisms and also associated with frontal gait disorders that also have FOG.

SCOPA-cog: cognitive rating scale used in European trials to track cognitive function in Parkinson disease patients.

Stroop test: test of mental flexability and set shifting using colored words that FOG patients have been shown in some preliminary studies to have more difficulty with than PD without FOG patients.

### Psychometric, sleep and quality of life scales

Epworth sleepiness scale: scale commonly used in sleep medicine to evaluate daytime sleepiness in patients due to untreated (or undertreated) sleep apnea. As Modafinil has benefit in arousal, will track patients to see if they are reporting improved daytime sleepiness which could account for improved FOG if it occurred.

REM sleep behavior disorder (RBD) screening questionaire: parasomnias are commonly reported in PD and RBD is a prominent non-motor feature that is thought to be a pre-motor symptoms of the disease. As reticular activating pathways play a role in sleep, this will be used as measure to see if sleep is altered by Modafinil through its putative role in the reticular activating system.

Apathy evaluation scale (AES): Validated questionnaire to assess apathy in patients. Apathy has been reported as correlated with overall level of motor function and will be tracked to see if apathy improved with the treatment.

PDQ-39: validated questionnaire to assess overall quality of life in PD patients and will be tracked to determine whether any objective improvement in FOG overall leads to improved subjective assessment of quality of life.

### Other measures

Schwab and England Activities of Daily Living Scale: subjective global percentage assessment of overall function of PD patients that will be tracked over the trial.

Hoehn and Yahr scale staging of Parkinson’s disease: staging scale, primarily developed for research based on unilateral, bilateral, postural stability and level of gait disability which will be tracked and requires no addition patient time, other than the UPDRS motor score above.

# ADVERSE EVENTS

## Experimental Therapy

While Modafinil does not have an indication in the treatment of freezing of gait in Parkinson disease it is FDA approved for the treatment of narcolepsy and has had a number of small clinical trials for use on various psychiatric conditions (ex. Depression and PTSD) but also is commonly used off label for daytime sleepiness. It has also been previously tested in Parkinson’s disease patients with fatigue (total of 53 patients).^19, 20^ For the most recent safety update, please refer to the commercially available drug insert for Modafinil (PROVIGIL).

Modafinil has previously been FDA approved for use in patients with narcolepsy. In clinical trials 151 protocol specified doses of up to 1600 mg/day have been administered to 32 subjects with no reported life-threatening events. Case reports of up to 4500 mg/dose also have not reported life-threatening events but reported excitation, agitation, insomnia and moderate elevation in hemodynamic parameters.

### Contraindications:

- Allergic reaction to any of the ingredients
- Have had a rash or allergic reaction to either Modafinil or armodafinil

### Special Warnings and Precautions for Use

### Modafinil is a controlled substance as it can be abused and/or lead to dependence. Keep in a safe place to avoid misuse and abuse.

### In-vitro data suggests that Modafinil is a weak inducer of CYP3A activity in a concentration dependent manner and therefore may reduce the effectiveness of drugs that are substrates for CYP3A enzymes (e.g. steroidal contraceptives, cyclosporine, midazolam, triazolam). Data also suggest that Modafinil is a weak inducer of CYP1A2, CYP2B6, and CYP2C9 (warfarin and phenytoin). Modafinil is also a reversible inhibitor of CYP2C19 and therefore may increase concentrations of drugs metabolized by this enzyme (e.g. phenytoin, diazepam, propranolol, omeprazole and clomipramine). Modafinil may interact with some CNS active drugs that PD patients may be using including certain tricyclic antidepressants (TCAs) (metabolized by CYP2C19) and selective serotonin uptake inhibitors (SSRIs) (metabolized by CYP2D6). Also concomitant administration of quetiapine reduced the systemic exposure of quetiapine. A recent report of a large overdose did not result in serious harmful effects.^21^

### Adverse Events:

### The following serious adverse reactions have been reported in the FDA approved label for Modafinil and will be monitored for including:

### serious rash (0.8%) including Stevens-Johnson Syndrome

### Angioedema and Anaphylaxis (one case reported out of 1595 patients)

### multi-organ hypersensitivity reactions

### persistent sleepiness

### psychiatric symptoms (0.3% including: mania, delusions, suicidal ideations, paranoid delusions, auditory hallucinations with doses of 600 mg/day)

### effect on ability to drive and use Machinery

### cardiovascular events (chest pain, palpitations, dyspnea, ischemic T-wave changes on electrocardiogram [occurred in 3 subjects with mitral valve prolapse or left ventricular hypertrophy], one episode of 9-seconds of asystole in a 35 year-old obese narcoleptic male with a prior history of syncope on 300 mg/day modafinil)

The following adverse reactions were reported in placebo controlled trials of Modafinil in Narcolepy, obstructive sleep apnea and shift work disorder in 1% or more of the modafinil treated group.

|  | modafinil (%) (n=934) | placebo (%) (n=567) |
| --- | --- | --- |
| Headache | 34 | 23 |
| Nausea | 11 | 3 |
| Nervousness | 7 | 3 |
| Rhinitis | 7 | 6 |
| Back Pain | 6 | 5 |
| Diarrhea | 6 | 5 |
| Anxiety | 5 | 1 |
| Dizziness | 5 | 4 |
| Dyspepsia | 5 | 4 |
| Insomnia | 5 | 1 |
| Anorexia | 4 | 1 |
| Dry Mouth | 4 | 2 |
| Pharyngitis | 4 | 2 |
| Chest Pain | 3 | 1 |
| Hypertension | 3 | 1 |
| Abnormal Liver Function | 2 | 1 |
| Constipation | 2 | 1 |
| Depression | 2 | 1 |
| Palpitation | 2 | 1 |
| Paresthesia | 2 | 0 |
| Somnolence | 2 | 1 |
| Tachycardia | 2 | 1 |
| Vasodilatation | 2 | 0 |
| Abnormal Vision | 1 | 0 |
| Agitation | 1 | 0 |
| Asthma | 1 | 0 |
| Chills | 1 | 0 |
| Confusion | 1 | 0 |
| Dyskinesia | 1 | 0 |
| Edema | 1 | 0 |
| Emotional Lability | 1 | 0 |
| Eosinophilia | 1 | 0 |
| Epistaxis | 1 | 0 |
| Flatulence | 1 | 0 |
| Hyperkinesia | 1 | 0 |
| Hypertonia | 1 | 0 |
| Mouth Ulceration | 1 | 0 |
| Sweating | 1 | 0 |
| Taste Perversion | 1 | 0 |
| Thirst | 1 | 0 |
| Tremor | 1 | 0 |
| Urine Abnormality | 1 | 0 |
| Vertigo | 1 | 0 |

## Adverse Event Monitoring

Adverse event data collection and reporting, which are required as part of every clinical trial, are done to ensure the safety of Subjects enrolled in the studies as well as those who will enroll in future studies using similar agents. Adverse events are reported in a routine manner at scheduled times during a trial. Additionally, certain adverse events must be reported in an expedited manner to allow for optimal monitoring of subject safety and care.

## Definitions

### Definition of Adverse Event

An adverse event (AE) is any untoward medical occurrence in a subject receiving study treatment and which does not necessarily have a causal relationship with this treatment. An AE can therefore be any unfavorable and unintended sign (including an abnormal laboratory finding), symptom, or disease temporally associated with the use of an experimental intervention, whether or not related to the intervention.

### Severity of Adverse Events

The severity of adverse events will be graded as follows:

Mild: the event causes discomfort without disruption of normal daily activities.

Moderate: the event causes discomfort that affects normal daily activities.

Severe: the event makes the patient unable to perform normal daily activities or significantly affects his/her clinical status.

### Serious Adverse Events

A “serious” adverse event is defined in regulatory terminology as any untoward medical occurrence that meets one or more of the following criteria:

#### Results in death.

If death results from (progression of) the disease, the disease should be reported as event (SAE) itself.

#### Is life-threatening.

(the patient was at risk of death at the time of the event; it does not refer to an event that hypothetically might have caused death if it were more severe).

#### Requires in-patient hospitalization or prolongation of existing hospitalization for ≥ 24 hours.

#### Results in persistent or significant disability or incapacity.

#### Is a congenital anomaly/birth defect

#### Is an important medical event

#### Any event that does not meet the above criteria, but that in the judgment of the investigator jeopardizes the patient, may be considered for reporting as a serious adverse event. The event may require medical or surgical intervention to prevent one of the outcomes listed in the definition of “Serious Adverse Event“.

For example: allergic bronchospasm requiring intensive treatment in an emergency room or at home; convulsions that may not result in hospitalization; development of drug abuse or drug dependency.

## Steps to Determine If an Adverse Event Requires Expedited Reporting

Step 1: Identify the adverse event.

Step 2: Determine whether the adverse event is related to the protocol therapy.

Attribution categories are as follows:

- - Definite – The AE *is clearly related* to the study treatment.
  - Probable – The AE *is likely related* to the study treatment.
  - Possible – The AE *may be related* to the study treatment.
  - Unrelated – The AE *is clearly NOT related* to the study treatment.

Step 3: Determine the prior experience of the adverse event.

Expected events are those that have been previously identified as resulting from administration of the agent. An adverse event is considered unexpected, for expedited reporting purposes only, when either the type of event or the severity of the event is not listed in:

- the current known adverse events listed in the Agent Information Section of this protocol;
- the drug package insert;

Step 4: Determine whether the adverse event is a Serious Adverse Event

## Reporting Requirements for Adverse Events

### Expedited Reporting

- The Principal Investigator must be notified within 24 hours of learning of any serious adverse events, regardless of attribution, occurring during the study.
  - - The IRB must be notified within 10 business days of any unanticipated problems involving risk to subjects or others” (UPIRTSO). A UPIRTSO is defined as any problem, event or new information that is:

1. Unanticipated or unexpected;
2. Related to the research; and
3. Involves new or increased risks to subjects or others.
   - - The FDA should be notified within 7 business days of the PI learning of any unexpected fatal or life-threatening adverse event with possible relationship to study drug, and 15 business days of the Sponsor learning of any event that is considered: 1) serious, 2) unexpected, and 3) at least possibly related to study participation.

### Routine Reporting

- All other adverse events- such as those that are expected, or are unlikely or definitely not related to the study participation- are to be reported annually as part of regular data submission.

## Unblinding Procedures

Depending upon the AE or if there is a SAE or unforeseen event, the treatment of the subject may need to be unblinded as part of management of the event at the discretion of the PI. The IRB will be notified within 10 days of the unblinding and it will be documented in the subject’s source document.

## Stopping Rules

Dosing of a particular subject may be stopped prematurely by the principle investigator if the subject:

1. Experiences an intolerable adverse event (see section 6.3);

2. Develops a clinically significant laboratory or ECG abnormality;

3. Develops an allergic or anaphylactoid reaction to the medication;

4. The principal investigator consider that the number and/or severity of adverse events justify discontinuation of the study

# STATISTICAL CONSIDERATIONS

The study is a prospective repeated measures longitudinal design. The primary outcome response is the FOG-Q total score and the stride length changes, and the secondary responses are the UPDRS Part III subscale, the PDQ-39, the RBD scale, and the Epworth Sleepiness Scale. These measures will be observed at 6 time points for both study arms. A primary statistical goal is to estimate the mean response at all six time points for each group and subsequently estimate changes in the outcome measures for each study group from baseline. Additionally, it is of interest to estimate the how the study groups differ in their mean change from baseline. A two group repeated measures linear model will be employed to determine significance for within each group and then to compare among the groups. Significance will be declared at the 5% level of significance and Bonferroni methods will be used for multiple comparisons. This analysis approach will be applied to the primary and secondary responses.

The study calls for 10 subjects in both arms for a total of 20 patients. Within a study arm the linear model will have in excess of 80% power to detect a 1.75 unit difference in observed means. Based on standard deviation estimates from previous researchers in similar settings,^22^ the FOG-Q (the primary response) has a standard deviation of 3.58. This implies that we will have power to detect a 6.265 (1.75*3.58) unit difference among time point means at the 5% level of significance. Power calculations are from ‘Tables of Sample Sizes in the Analysis of Variance’ published by Bratcher.^23^

# STUDY MANAGEMENT

## Institutional Review Board (IRB) Approval and Consent

This study will be conducted in accordance with all applicable government regulations and University of Arkansas for Medical Sciences research policies and procedures.  This protocol and any amendments will be submitted and approved by the UAMS Institutional Review Board (IRB).

The formal consent of each subject, using the IRB-approved consent form, will be obtained before that subject is submitted to any study procedure.  All subjects for this study will be provided a consent form describing this study and providing sufficient information in language suitable for subjects to make an informed decision about their participation in this study.  The person obtaining consent will thoroughly explain each element of the document and outline the risks and benefits, alternate treatment(s), and requirements of the study.  The consent process will take place in a quiet and private room, and subjects may take as much time as needed to make a decision about their participation.  Participation privacy will be maintained and questions regarding participation will be answered.  No coercion or undue influence will be used in the consent process.  This consent form must be signed by the subject or legally acceptable surrogate, and the individual obtaining the consent.  A copy of the signed consent will be given to the participant, and the informed consent process will be documented in each subject’s research record.

The trial will be registered on clinicaltrials.gov in accordance with current guidelines.

## Data Management and Monitoring/Auditing

Paper data will be stored in a locked file cabinet in the PI’s (Dr. Virmani’s) office in the Spine building. Additionally, source paper documents will be routinely scanned and the electronic versions will be stored on a secure computer located in the gait lab, with access only to the PI, co-Investigators, other members of the research team and safety monitors including the medical monitor and the UAMS Office of Research Regulatory Affairs (ORRA) after IRB approval. Data auditing will be at the discretion of the UAMS IRB committee as needed.

## Adherence to the Protocol

Except for an emergency situation in which proper care for the protection, safety, and well-being of the study patient requires alternative treatment, the study shall be conducted exactly as described in the approved protocol. Investigators may only implement a deviation from or a change to the protocol to eliminate an immediate hazard(s) to subjects without prior IRB approval.

## Amendments to the Protocol

Should amendments to the protocol be required, the amendments will be originated and documented by the Principal Investigator. It should also be noted that when an amendment to the protocol substantially alters the study design or the potential risk to the patient, a revised consent form might be required.

The amended protocol, and if required the amended consent form, must be approved by the IRB prior to implementation.

## Record Retention

Study documentation includes all Case Report Forms, data correction forms or queries, source documents, monitoring logs/letters, and regulatory documents (e.g., protocol and amendments, IRB correspondence and approval, signed patient consent forms). All study records will be retained in accordance with applicable institutional and applicable regulatory requirements.

## Dissemination of data

Results of this study may be used for presentations, posters, or publications. The publications will not contain any identifiable information that could be linked to a participant but can contain sample videos after the use of video editing software to mask identifying facial features.

## Obligations of Investigators

The Principal Investigator is responsible for the conduct of the clinical trial at the site in accordance with applicable regulatory requirements. The Principal Investigator is responsible for personally overseeing the treatment of all study subjects. The Principal Investigator must assure that all study site personnel, including sub-investigators and other study staff members, adhere to the study protocol and all applicable regulations and guidelines regarding clinical trials both during and after study completion.

The Principal Investigator will be responsible for assuring that all the required data will be collected and entered onto the Case Report Forms and these will be reviewed continually. Periodically, monitoring visits will be conducted by ORRA monitors and the Principal Investigator will provide access to his/her original records to permit verification of proper entry of data. At the completion of the study, all case report forms will be reviewed by the Principal Investigator and will require his/her final signature to verify the accuracy of the data.

# REFERENCES

1. Fahn S. The freezing phenomenon in parkinsonism. Advances in neurology 1995;67:53-63.

2. Nutt JG, Bloem BR, Giladi N, Hallett M, Horak FB, Nieuwboer A. Freezing of gait: moving forward on a mysterious clinical phenomenon. Lancet neurology 2011;10:734-744.

3. Virmani T, Moskowitz CB, Vonsattel JP, Fahn S. Clinicopathological characteristics of freezing of gait in autopsy-confirmed Parkinson's disease. Movement disorders : official journal of the Movement Disorder Society 2015;30:1874-1884.

4. Adkin AL, Frank JS, Jog MS. Fear of falling and postural control in Parkinson's disease. Movement disorders : official journal of the Movement Disorder Society 2003;18:496-502.

5. Grimbergen YA, Munneke M, Bloem BR. Falls in Parkinson's disease. Current opinion in neurology 2004;17:405-415.

6. Kerr GK, Worringham CJ, Cole MH, Lacherez PF, Wood JM, Silburn PA. Predictors of future falls in Parkinson disease. Neurology 2010;75:116-124.

7. Espay AJ, Fasano A, van Nuenen BF, Payne MM, Snijders AH, Bloem BR. "On" state freezing of gait in Parkinson disease: a paradoxical levodopa-induced complication. Neurology 2012;78:454-457.

8. Volkow ND, Wang GJ, Fowler JS, et al. Dopamine transporter occupancies in the human brain induced by therapeutic doses of oral methylphenidate. The American journal of psychiatry 1998;155:1325-1331.

9. Moreau C, Delval A, Defebvre L, et al. Methylphenidate for gait hypokinesia and freezing in patients with Parkinson's disease undergoing subthalamic stimulation: a multicentre, parallel, randomised, placebo-controlled trial. Lancet neurology 2012;11:589-596.

10. Garcia-Rill E, Hyde J, Kezunovic N, Urbano FJ, Petersen E. The physiology of the pedunculopontine nucleus: implications for deep brain stimulation. Journal of neural transmission 2015;122:225-235.

11. Mazzone P, Lozano A, Stanzione P, et al. Implantation of human pedunculopontine nucleus: a safe and clinically relevant target in Parkinson's disease. Neuroreport 2005;16:1877-1881.

12. Mazzone P, Vilela Filho O, Viselli F, et al. Our first decade of experience in deep brain stimulation of the brainstem: elucidating the mechanism of action of stimulation of the ventrolateral pontine tegmentum. Journal of neural transmission 2016.

13. Boucetta S, Cisse Y, Mainville L, Morales M, Jones BE. Discharge profiles across the sleep-waking cycle of identified cholinergic, GABAergic, and glutamatergic neurons in the pontomesencephalic tegmentum of the rat. The Journal of neuroscience : the official journal of the Society for Neuroscience 2014;34:4708-4727.

14. Kezunovic N, Urbano FJ, Simon C, Hyde J, Smith K, Garcia-Rill E. Mechanism behind gamma band activity in the pedunculopontine nucleus. The European journal of neuroscience 2011;34:404-415.

15. Gerrard P, Malcolm R. Mechanisms of modafinil: A review of current research. Neuropsychiatric disease and treatment 2007;3:349-364.

16. Garcia-Rill E, Heister DS, Ye M, Charlesworth A, Hayar A. Electrical coupling: novel mechanism for sleep-wake control. Sleep 2007;30:1405-1414.

17. Urbano FJ, Leznik E, Llinas RR. Modafinil enhances thalamocortical activity by increasing neuronal electrotonic coupling. Proceedings of the National Academy of Sciences of the United States of America 2007;104:12554-12559.

18. Volkow ND, Fowler JS, Logan J, et al. Effects of modafinil on dopamine and dopamine transporters in the male human brain: clinical implications. JAMA 2009;301:1148-1154.

19. Lou JS, Dimitrova DM, Park BS, et al. Using modafinil to treat fatigue in Parkinson disease: a double-blind, placebo-controlled pilot study. Clin Neuropharmacol 2009;32:305-310.

20. Ondo WG, Fayle R, Atassi F, Jankovic J. Modafinil for daytime somnolence in Parkinson's disease: double blind, placebo controlled parallel trial. Journal of neurology, neurosurgery, and psychiatry 2005;76:1636-1639.

21. Carstairs SD, Urquhart A, Hoffman J, Clark RF, Cantrell FL. A retrospective review of supratherapeutic modafinil exposures. J Med Toxicol 2010;6:307-310.

22. Shine JM, Moore ST, Bolitho SJ, et al. Assessing the utility of Freezing of Gait Questionnaires in Parkinson's Disease. Parkinsonism & related disorders 2012;18:25-29.

23. Bratcher TL, Moran MA, Zimmer WJ. Tables of Sample Sizes in the Analysis of Variance. Journal of Quality Technology 1970;2:156-164.
