# Supplementary material for: Lessons Learned From a Delayed‐Start Trial of Modafinil for Freezing of Gait in Parkinson's Disease
Source: Ann Clin Transl Neurol. 2025 Dec 8;13(5):899–910. doi: 10.1002/acn3.70276 (PMC13161890; doi:10.1002/acn3.70276)
Supplement: Supplementary file 2 — Table S1: Spatiotemporal gait and turn parameters—sources and definitions. Table S2: All adverse events reported during the first 12 weeks in participants completing at least one post‐randomization visit. Table S3: Quantified Total and Percent Freeze Time of all participants who completed Visit 3. [file ACN3-13-899-s001.docx]

| **Table S1** – Spatiotemporal gait and turn parameters – sources and definitions | | |
| --- | --- | --- |
| Parameter | Source | Definition |
| Spatiotemporal gait parameters: | | |
| Foot-strike-length^1,2^ (cm) | PKMAS | length of the major axis of the ellipse enclosing each footstep; PKMAS creates an ellipse around each footstep during footstep identification. |
| Foot-strike-area^1,2^ (cm^2^) | PKMAS | the area of the ellipse using the major and minor axes as the radii. |
| Integrated-pressure^1,2^ (pressure x s) | PKMAS | the sum of pressure applied by a footstep at each sampling time (120 Hz sampling rate) in the area of its contact with the ground. |
| Step-length^1^ (cm) | PKMAS | the distance between heel strikes of two consecutive footsteps, i.e., right to left or left to right heel strikes. |
| Stride-length^1,2^ (cm) | PKMAS | the distance between heel strikes of two consecutive footsteps of the same foot, i.e., two right or two left heel strikes. |
| Stride-width^1^ (cm) | PKMAS | the perpendicular distance between an imaginary line connecting the two consecutive heel contacts of the same foot and the distance between the heel contact of the other foot that happens in between this event in a gait cycle. For example, a left-right-left gait cycle where the stride width is the perpendicular distance between the two left heels and the middle right heel. |
| Step-time^1^ (s) | PKMAS | the time difference (s) between the initial heel contacts with the mat of two consecutive footsteps of alternating feet, i.e., right to left or left to right. |
| Stride-time^1,2^ (s) | PKMAS | the time difference (s) between the initial heel contacts with the mat of two consecutive footsteps of the same foot, i.e., two right or two left. |
| Gait-cycle-time^1^ (s) | PKMAS | Same as stride-time above |
| Stride-velocity^1,2^ (cm/s) | PKMAS | the stride-length divided by stride-time, calculated for each gait cycle. |
| Stance-time^1,2^ (s) | PKMAS | the time difference between the first and last contact of each footstep plus the sampling time and represents the time duration of foot contact with the ground. |
| Swing-time^1,2^ (s) | PKMAS | the difference between gait cycle time and stance time and represents the time duration when the foot is off the ground. |
| Stance-percent^1,2^ | PKMAS | a percentage measure of time spent in stance phase of the gait cycle and calculated as stance-time/gait-cycle-time. |
| Swing-percent^1,2^ | PKMAS | a percentage measure of time spent in swing phase of the gait cycle and calculated as swing-time/gait cycle time. |
| Single-support-time^1^ (s) | PKMAS | the time when only the footstep being measured is in contact with the ground, calculated as applicable for each footstep. |
| Total-double-support-time^1^ (s) | PKMAS | the total time when both feet are in contact with the ground during stance phase of a footstep being measured, calculated as applicable for each footstep. |
| Single-support-percent^1^ | PKMAS | a percentage measure of time spent in single-support phase of the gait cycle and calculated as single-support-time/gait-cycle-time. |
| Total-double-support-percent^1^ | PKMAS | a percentage measure of time spent in total-double-support phase of the gait cycle and calculated as total-double-support-time/gait- cycle-time. |
| Stance-COP-distance^1,2^ (cm) | PKMAS | stance Center of Pressure distance; the Pythagorean distance between the first and last contact points of the Center Of Pressure (COP) waveform trail for a footstep in stance phase, based on X and Y coordinates corresponding to first and last contact times of a footstep during stance. |
| Single-support-COP-distance^1^ (cm) | PKMAS | this measure is similar to stance-COP-distance defined above except that the waveform trail measured is from the first to last instances of only the single-support part of the footstep |
| Ambulation time (s) | PKMAS | an overall measure of the walk trial calculated as a summation of time of entire walk trial consisting of initial contact of first to initial contact of last footsteps of each walk segment |
| Cadence^1^ (steps/minute) | PKMAS | an overall measure of the walk trial calculated as total footsteps (excluding 1)/ ambulation-time |
| Velocity^1^ (cm/s) | PKMAS | an overall measure of the walk trial calculated as the sum of all stride-lengths (both feet)/ sum of all stride-time (both feet) |
| Additional turn specific parameters: | | |
| Turn-time^2^ (s) | author defined | the difference in time^#^ between the first contact of the last normal-angled, pre-turn footstep, and the last contact of the first normal-angled, post-turn footstep. |
| Turn-length^2^ (cm) | author defined | the distance (difference) between the maximum and minimum value of the X coordinates^#^ of foot placement on the mat. |
| Turn-width^2^ (cm) | author defined | the distance (difference) between the maximum and minimum value of the Y coordinates^#^ of foot placement on the mat. |
| Turn-rectangular-area^2^ (cm^2^) | author defined | turn-length*turn-width |
| Turn-ellipse-area^2^ (cm^2^) | author defined | (π*[turn length/2]*[turn width/2]) |
| Turn-step-count^2^ (no.) | author defined | sum of right and left footsteps in each turn segment |
| Foot-strike-width^2^ (cm) | PKMAS | length of the minor axes of the ellipse enclosing each footstep |
| Foot-strike-length-percent^2^ | PKMAS | a percentage measure of foot length relative to the longest foot length measured for all selected footsteps, for each foot. |
| Timed-Up-and-Go (TUG) specific parameters: | | |
| Sit-to-stand-time^3^ (s) | author defined | the time taken to completely rise from the chair before starting to walk and includes any postural adjustment while trying to rise from the chair until starting to walk. |
| TUG-turn-time^3^ (s) | author defined | the time taken to complete the turn at the free end of the mat and does not include the time taken to walk the lengths of the mat pre- and post-turn. |
| TUG-turn-cadence^3^ (steps/s) | author defined | calculated as the number of steps taken to complete the TUG turn/ TUG-turn-time |
| Stand-to-sit-time^3^ (s) | author defined | the time taken to completely settle in the chair at the end of the task from standing position and includes any positional adjustments/turn required to change direction when reaching the chair and sit back down |
| Stand-to-sit-cadence^3^ (steps/s) | author defined | calculated as the number of steps taken from initiating the maneuver to sit back down in chair at the end of TUG to actually completing it/ stand-to-sit-time |
| TUG-duration^3^ (s) | author defined | the total time taken to complete one iteration of the TUG task |

Subjects wore their preferred, comfortable footwear.

All “PKMAS” calculations were auto-generated by the PKMAS software and definitions based on our understanding of the PKMAS Measurements and Definitions manual Protokinetics provided with the software

^@^ PKMAS definition altered to represent that this was auto-generated by PKMAS for turn segments

^#^ time stamps and mat location coordinates for each footstep were auto-generated by PKMAS

all “author defined” turn parameter calculations were done in Microsoft Excel.

^1^single-task and dual-task gait parameter

^2^turn parameter

^3^timed-up-and-go (TUG) parameter

| **Table S2: All adverse events reported during the first 12 weeks in participants completing at least one post-randomization visit.** | | | | | | | |
| --- | --- | --- | --- | --- | --- | --- | --- |
| **Body System** | **Description of SE** | **Early-start (n=12)** | **Delayed-start (n=7)** | **Body System** | **Description of SE** | **Early-start (n=12)** | **Delayed-start (n=7)** |
| cardiovascular | LE edema |  | 1 | Neurologic | paresthesias | 1 | 1 |
|  | dyspnea on exertion |  | 1 |  | morning grogginess |  | 1 |
|  | palpitations |  | 1 |  | daytime sleepiness | 1 | 1 |
|  | lightheadedness on standing | 1 |  |  | insomnia | 1 | 3 |
| dermatologic | dry skin | 1 |  |  | falls | 1 | 2 |
|  | facial rash | 1 |  |  | sciatica |  | 1 |
|  | skin tag | 1 |  |  | daytime confusion | 1 |  |
| endocrine | cold intolerance |  | 1 |  | dysphagia | 1 |  |
|  | diagnosed with diabetes | 1 |  |  | fatigue | 1 |  |
| general | Rhinorrhea | 2 | 2 |  | hallucinations | 1 |  |
|  | allergy flare |  | 1 |  | hand cramps | 1 |  |
| eye | lower eyelid edema | 1 |  |  | freezing of gait | 1 |  |
| ear/labrynth | tinnitus | 1 |  |  | headaches | 2 | 1 |
| Gastrointestinal | abdominal pain |  | 1 |  | imbalance | 2 |  |
|  | constipation |  | 1 |  | difficulty getting out of bed | 1 |  |
|  | nausea | 1 |  |  | difficulty with ADLs | 1 |  |
| metabolism | hyperphagia |  | 1 |  | slowed gait | 1 |  |
| musculoskeletal | calf pain/cramps | 2 | 2 |  | toe walking | 1 |  |
|  | bursitis/epicondylitis |  | 1 |  | word finding difficulty | 1 |  |
|  | neck stiffness | 1 |  | psychiatric | anxiety | 1 | 2 |
| ophthalmic | lacrimation | 1 | 1 |  | depressed mood | 1 |  |
|  | visual floaters/flashing | 1 | 1 |  | irritability | 1 |  |
|  | blurred vision | 1 |  | infectious | urinary tract infection | 1 |  |
| renal /urinary | nocturia | 4 | 1 |  |  |  |  |
|  | nephrolithiasis | 1 |  |  |  |  |  |
|  | urinary incontinence | 1 |  | **Any Side Effect** |  | **12** | **7** |

| **Table S3: Quantified Total and Percent Freeze Time of all participants who completed Visit 3.** | | | | | | | | |
| --- | --- | --- | --- | --- | --- | --- | --- | --- |
| **Sub ID** | **Total Freeze Time (s) (FT)** | | | | **Percent Freeze Time (%) (%FT)** | | | |
|  | **Visit 1**  **0 weeks** | **Visit 3**  **12 weeks** | **Visit 5**  **24 weeks** | **Visit 6**  **26 weeks** | **Visit 1**  **0 weeks** | **Visit 3**  **12 weeks** | **Visit 5**  **24 weeks** | **Visit 6**  **26 weeks** |
| Early-start | | | | | | | | |
| 1 | 18.9 | 13.6 | 44.3 | 91.0 | 17% | 6% | 29% | 56% |
| 2 | 0.0 | 0.0 | 0.0 | 0.0 | 0% | 0% | 0% | 0% |
| 3 | 20.7 | 262.0 | - | - | 15% | 65% | - | - |
| 4 | 0.0 | 90.7 | 67.0 | 86.1 | 0% | 39% | 36% | 45% |
| 5 | 2.1 | 0.0 | 0.0 | 1.5 | 4% | 0% | 0% | 3% |
| 6 | 124.1 | 49.7 | 93.2 | 57.1 | 59% | 36% | 62% | 43% |
| 7 | 57.5 | 29.1 | 35.6 | 52.8 | 35% | 24% | 22% | 28% |
| 8 | 4.9 | 1.5 | 2.2 | 16.1 | 4% | 1% | 2% | 10% |
| 9 | 0.0 | 0.0 | 0.0 | 0.0 | 0% | 0% | 0% | 0% |
| 10 | 45.7 | 82.2 | 31.1 | 26.4 | 31% | 45% | 22% | 18% |
| 11 | 4.1 | 6.5 | 4.9 | 17.7 | 7% | 11% | 8% | 27% |
| 12 | 12.6 | 6.0 | 11.3 | 19.5 | 15% | 8% | 13% | 19% |
| Delayed-start | | | | | | | | |
| 13 | 49.3 | 125.5 | 83.4 | 89.5 | 38% | 71% | 62% | 63% |
| 15 | 0.0 | 0.0 | 0.0 | 0.0 | 0% | 0% | 0% | 0% |
| 16 | 0.0 | 0.0 | 0.0 | 0.0 | 0% | 0% | 0% | 0% |
| 17 | 6.7 | 1.0 | 0.8 | 7.0 | 6% | 1% | 1% | 6% |
| 18 | 7.9 | 2.2 | 4.7 | 6.2 | 11% | 3% | 6% | 9% |
| 20 | 35.6 | 11.6 | - | - | 36% | 17% | - | - |
| 21 | 17.3 | 0.0 | 12.5 | 12.4 | 23% | 0% | 20% | 21% |
|  | | | | | | | | |
